# Observed Changes in the Frequency, Intensity, and Spatial Patterns of Nine Natural Hazards in the United States from 2000 to 2019

**DOI:** 10.3390/su14074158

**Published:** 2022-03-31

**Authors:** J. K. Summers, A. Lamper, C. McMillion, L. C. Harwell

**Affiliations:** United States Environmental Protection Agency, Office of Research and Development, Center for Measurements and Modeling, Gulf Breeze, FL 32561, USA;

**Keywords:** climate, natural hazards, resilience, time series

## Abstract

There is increasing evidence from across the globe that climate change results in changes in the frequency, location, and impact of natural hazards. Much of this evidence is conceptual, inferential, or simply assumed. To provide objective support to confirm these hypotheses, we constructed county-level time-series datasets (2000–2019) for nine natural hazards for the entire United States. Hazards considered for this study included hurricanes, tropical storms, landslides, wildfires, earthquakes, drought, inland flooding, coastal flooding, and tornadoes. Geospatial analysis techniques were used to calculate the percentage (range: 0–100) of land area in each county exposed to each natural hazard for all the years that hazard data were available. The best available data were acquired from publicly accessible sources. Cumulative distribution functions were calculated for each hazard in five-year intervals to test for statistically significant changes in distribution patterns across the five-year time periods using the Kolmogorov–Smirnov test. There were significant changes in hurricanes, tropical storms, and drought over the two decades; changes in tornadoes, landslides, and wildfires were not significant in terms of frequency, likely due to the site-specific nature of their occurrences. The intensity and spatial distribution and an emerging hot spot and spatial trend analyses and an emerging hot spot and spatial trend analyses were also completed (except for flooding events and earthquakes due to insufficient data). All datasets provide empirical support for earlier inferences concerning the connections between the hazards and climate change. Analyses showed apparent changes in the frequency and intensity of hurricanes, tropical storms, and drought-related to climate change factors. Internal and coastal flooding also demonstrated these connections, although the length of the dataset did not permit significant testing but shows significant hot spots and trending locations. Tornadoes, landslides, and wildfires showed significant hot spots and trending locations, but the specific locational nature of the data did not show significant changes in frequency. Earthquakes showed no significant changes over the time period.

## 1. Introduction

Increasing evidence suggests that climate change impacts natural hazard events already being observed across the globe. Using a machine-learning language model to identify documentation of observed climate impacts, Callaghan et al. [1] estimated that over 100,000 publications have addressed a range of impacts. These events translate to about 85% of the world’s population being affected by climate change [2]. Callaghan et al. [1] literature analysis provided corroboration of the lived experiences of people from Africa to North America to Asia. Studies directly examining these hazards’ frequency, intensity, and spatial location collectively are few, primarily because they require extensive databases to reveal patterns and trends.

There is increasing evidence from across the globe that climate change results in changes in the frequency, location, and impact of natural hazards. Much of this evidence is conceptual, inferential, or simply assumed. To provide objective support to confirm these hypotheses, we constructed county-level time-series datasets (2000–2019) for nine natural hazards for the entire United States. Hazards considered for this study included hurricanes, tropical storms, landslides, wildfires, earthquakes, drought, inland flooding, coastal flooding, and tornadoes. Mechanisms have been proposed to explain the potential for increases in several natural hazards and climate changes [3–6]. Some natural hazards are easily related to climate change (e.g., hurricanes, flooding), others (e.g., earthquakes) are not as easily mechanistically connected to climate change (likely more geological). In contrast, the connection of others (e.g., landslides) to climate change lies somewhere in between geomorphological and climate factors. This manuscript addresses all three categories of natural hazards. Throughout this manuscript, changes in frequency, intensity, and spatial location of natural hazards are related to climate changes such as temperature changes and variability, rainfall variation, changes in wind patterns, and other specific instances of climate change.

The frequency of intense hurricanes will likely increase with anthropogenic climate change [7,8]. Modeling results have suggested that increases are substantial, approaching a doubling in frequency of severe category four and five hurricanes for each C in global warming [8–10]. Increases in hurricane wind hazards along the eastern coastline of the United States and the suggestion that even more significant interannual and intra-annual variations in hurricane frequencies can occur due to climate change have been suggested [11,12]. Estrada et al. [13] identified an upward trend in economic losses from 1900 to 2005 that commonly used socioeconomic variables cannot explain. Based on geophysical data, they inferred an upward trend in both the number and intensity of hurricanes in the North Atlantic basin consistent with the smoothed global average rise in surface air temperature.

It is important to consider the potential impacts of climate change on severe thunderstorms and tornadoes [14]. For example, researchers are unsure if tornadoes will become more frequent and stronger. Still, tornado outbreaks may become more damaging with increased temperatures modifying the weather in unexpected ways [15]. Therefore, examining the role of large-scale atmospheric circulation in creating favorable tornado environments could be a valuable approach to link climate change to the frequency, intensity, and lengthening of the season of future tornadoes [16].

Changes in climate to affect urban infrastructure through sea-level rise and increased frequency of flooding could be likely scenarios [17]. The potential impact of increased inland flooding due to climate change on the transportation networks in the Boston Metro Area was assessed using an urban transportation model [17]. While the modeling showed a doubling in delays and lost time associated with trips, the model assumed a positive relationship between global warming and inland flooding.

Traditional approaches used to design and operate urban storm drainage infrastructure have relied on past performance and the ability to extrapolate this performance of natural systems into the future. Due to the forecasted impact of climate change on weather patterns, it seems clear that designers and operators of storm drainage systems should prepare for greater uncertainty in the effectiveness of their storm drainage systems. A recent UK Government study considered the potential effects of climate and socio-economic change in the UK in terms of four future scenarios and their implications for the performance of existing storm drainage facilities [18,19]. Based on these scenarios, changes in the risk of flooding and the effectiveness of standard urban infrastructure responses were modeled, showing the potential for a thirty-fold increased flood risk, and traditional engineering measures likely would not provide adequate protection [20]. These modeling efforts assumed that climate change resulted in increased urban flooding.

The fire triangle must exist for a wildfire to start, which requires fuel, oxygen, and a heat source [21]. Climate change may increase the chances that all of these will be present. Proposed climate change-related rainfall anomalies can intensify drought in tropical and subtropical areas [22]. Rainfall tends to become more concentrated in winter, making other seasons, especially summer, hotter and drier [23,24]. Climate change is projected to boost wind energy due to enhanced differences in temperature between the land and the sea, resulting in more significant land-sea differences in air pressure [25]. Strong winds provide more oxygen for wildfires and encourage their spread, potentially outstripping firefighting capability [21]. The interplay between climate change and wildfires could be reinforcing and synergistic, with a profound impact on human health [26].

These studies (and most natural hazard-climate change literature) have in common that they are based on subjective or theoretical inference, qualitative modeling, or quantitative modeling. Few studies have examined recent data concerning the frequency, intensity, and location of natural hazards to determine if they are changing, making this approach novel in examining both natural hazards and climate factors. In 2017, the US Environmental Protection Agency (US EPA) published an index (Climate Resilience Screening Index—CRSI) to examine community resilience to twelve natural hazards [27–29]. The index included domains to document community risk or exposure, governance, societal attributes important to vulnerability and recovery, and information about the built and natural environments [27,30]. Drawing from the hazards described by Summers and colleagues [27,28,30], eight of the twelve hazards—hurricanes, landslides, internal floods, coastal floods, tornadoes, drought, earthquakes, and wildfires—and the addition of tropical storms, rounded out the nine hazards reviewed in this study. We examined land areas exposed to these natural hazards to assess changes in the temporal and spatial frequencies in five-year blocks over twenty years (2000–2019). This approach is novel in that it examines the national data at a county level and assesses changes in the overall trajectories of the data for these natural hazards. The approach relates the hazards to our understanding of climate change factors rather than using available information systems for real-time tracking of hazards using automated disaster analysis systems [31], the development of mobile communications networks for damage zones [32], or rapid mapping for flooding [33]. These assessments informed additional data analysis to investigate further hazard exposure measures and potential relationships to climate change. The approach (using “found” data for both statistical and spatial mapping) can be used to assess the associations of natural hazard occurrences, frequency, intensity, and spatial distribution at both smaller (communities and states) and larger (international and global) scales.

## 2. Methods

### Overall Approach:

**Figure 1.**
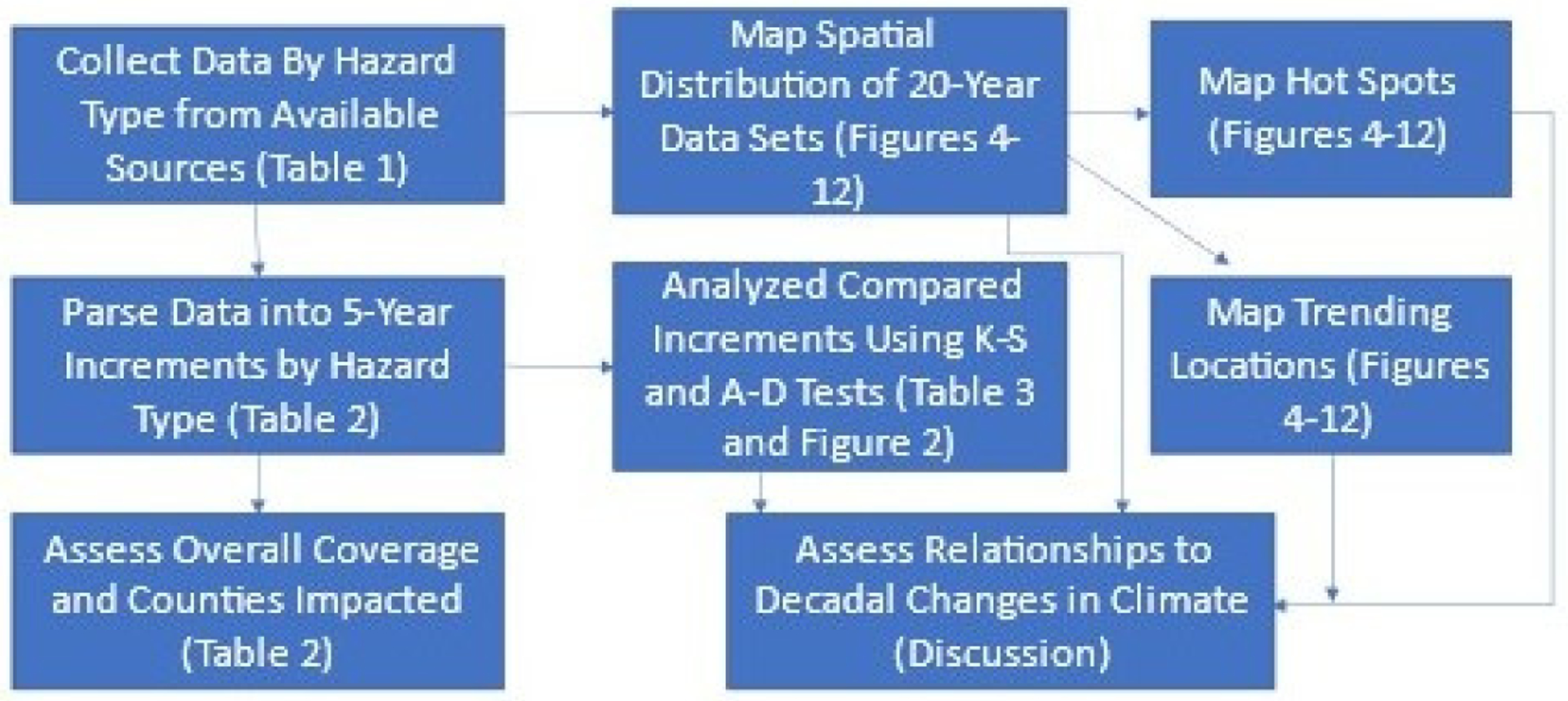
Workflow diagram for the overall process of data collection, analyses, and evaluation.

The methodology used to conduct these analyses examining changes in frequency, intensity, and spatial location is summarized in Figure 1 and described in detail below. The figure targets collecting data regarding natural hazards, parsing the data into analytical units, testing frequency changes, assessing spatial attributes (hot spots, trending locations), and accumulating evidence of climate change relationships.

### Database Development:

This dataset contains yearly natural hazard exposure estimates at the county-level for all 50 United States (US) and Puerto Rico (PR) across a 20-year timespan (2000–2019). Secondary data sources were used to collect both tabular and spatial hazard exposure information for each of the nine natural hazards included in this dataset (e.g., hurricanes, tropical storms, tornadoes, landslides, wildfires, drought, coastal and inland flooding, and earthquakes). Candidate secondary data were reviewed for accessibility, temporal and spatial scale, and data formatting. Data acceptance criteria were open access, per year basis, and vector or raster data format. When multiple data sources were available for the same natural hazard, datasets were compared, and the source most likely to continue publishing data was selected. In cases where data did not fully meet acceptance criteria, the best available data were used for further analysis. Hazard-specific secondary data source information is provided in Table 1. Each cell highlighted in grey either emphasizes a lack of temporal or spatial information (i.e., earthquake data were only available for three years).

### CDFs and Multiple Hazard Analysis:

Cumulative distribution functions (CDFs) were used to evaluate changes in the frequency of the nine examined natural hazards from 2000–2019. The cumulative distribution function (CDF) is a technique to describe the distribution of random variables. The advantage of the CDF is that it can be defined for any kind of variable (continuous, discrete, or mixed). The concept of the cumulative distribution function can be used in statistical analysis in two (similar) ways.

1. Cumulative frequency analysis is the analysis of the frequency of occurrence of values of a phenomenon less than a reference value. In these cases, data for cumulative distribution functions were estimated based on sample data weighted by county area and the number of occurrences of the natural hazard in that county in a given 5-year period.
2. The empirical distribution function is a formal direct estimate of the cumulative distribution function for which simple statistical properties (e.g., quartiles, median) can be derived and form the basis of various statistical hypothesis tests.

The Kolmogorov–Smirnov test was used to test whether CDFs based on 5-year periods between 2000 and 2019 were statistically different from one another. The Kolmogorov–Smirnov test is a non-parametric test based on cumulative distribution functions. It can be used to test whether two empirical distributions are different or whether an empirical distribution is different from an ideal distribution [34,35]. Various studies have found the Kolmogorov–Smirnov test is less powerful for testing normality than the Anderson–Darling test [36]. The Anderson–Darling test was also used to examine changes in frequency for six of the nine evaluated natural hazards.

Changes in the frequency of the observed nine natural hazards were also evaluated by examining the co-occurrence of hazards. Each of the four 5-year periods was quantified by the number of different hazards that occurred in a county. These multiple occurrences were examined for increases or decreases over the 20-year span.

### Spatial Analysis:

Natural hazard exposure estimates are represented as the percent area of county exposed to a particular hazard within a given year. To calculate these yearly exposure estimates, spatial data for each hazard (except drought) was brought into ArcGIS Pro version 2.6.3 for analysis. Despite a few deviations, the same workflow or sequence of tools was executed to calculate the percent area. In general terms, the workflow was as follows for each hazard:

1. Spatial data were downloaded, an ArcGIS Pro.aprx project file was created;
2. One map extent containing the most appropriate version of the Albers projection per each region (CONUS, AK, HI, and PR) was added to the project;
3. Hazard-specific shapefiles and the US Census Bureau’s 2018 county boundaries shapefile (https://www.census.gov/geographies/mapping-files/time-series/geo/carto-boundary-file.html, accessed on 15 January 2022) (were added to each map extent);
4. A definition query was written to limit data to only those events of interest and was then exported as a new shapefile;
5. The Buffer tool was run to mimic possible extent (if applicable);
6. Events were dissolved by year via the dissolve tool;
7. The Summarize-Within tool was then run (with group field parameter equal to “year”) for each map extent where events occurred, and,
8. Finally, output shapefiles and join tables from the Summarize-Within tool were exported as .csv files and brought into Microsoft Excel version 2102 to create the final dataset.

Within Excel, counties with no exposure to a particular hazard were assigned a value of 0, whereas counties with no data were assigned a value of *−*99.

Inputs for the tools listed in the workflow mentioned previously were unique to each hazard. Hurricane and tropical storm event data were downloaded as a polyline shapefile from NOAA’s IBTrACS site (Table 1). These data were limited to only those tracks occurring in the year 2000 or later and categorized as “main” or “provisional.” Similarly, tornado event data were downloaded as a polyline shapefile from NOAA’s SPC site and was limited to only those events occurring in the year 2000 or later with a magnitude of 0 or greater. Hurricanes, tropical storms, and tornadoes are the only hazards that necessitated the use of the Buffer tool. To estimate possible extent, hurricane and tropical storm events were buffered based on 34-knot wind radii quadrant fields. The four 34-knot wind radii quadrants or tropical-storm-force wind fields were chosen as the buffer’s starting point instead of the 50-knot or 64-knot quadrants because they illustrate the largest possible extent or area exposed to a hurricane. Finding an average value per category to represent the potential area of exposure was derived from FEMA’s NRI technical document (https://www.fema.gov/sites/default/files/documents/fema_national-risk-index_technical-documentation.pdf) (accessed on 15 January 2022). Tornado events were buffered based on a storm width field. The width value was converted from yards to associated map unit, meters and reported within the first field. Then, the width value converted to meters was divided by two and reported in the second field since the width represented the entire width of the event and not just storm center to edge. The buffer was based on this second field. The width field information is the only extent-related information provided by the NOAA dataset.

The Buffer tool provides a dissolve option within the tool itself, so each hazard’s remaining events were buffered and subsequently dissolved by year. Dissolving by year was necessary to ensure that percent area values were not over-represented due to overlapping events or errors in data entry (i.e., duplicate entries). The Summarize-Within tool was then used to calculate the percent of county area exposed to a given hazard within a particular year.

Wildfire events downloaded from the National Interagency Fire Center’s site and landslide events downloaded from USGS’s ScienceBase Catalog were represented as polygon features. They were limited to events occurring in or after the year 2000 (Table 1).

Landslide events were also limited to events with a confidence level of 3 or greater (Table 1). The remaining wildfire and landslide events were dissolved by year before executing the Summarize-Within tool.

Drought, coastal and inland flooding, and earthquakes are the hazards with the most notable procedural differences. Drought events from 2000–2020 with an intensity of D2 (severe drought) or greater were downloaded from the US Drought Monitor’s web database via an API data retrieval request URL in R version 3.6.0. Percent area values were calculated by averaging all weekly data within a county for each year (Table 1). In contrast to the previously mentioned hazards, data for coastal and inland flooding and earthquakes were not event-based. Instead these hazards were represented by the secondary data source’s latest data release, which is essentially a snapshot in time of possible exposure.

Coastal flood data from NOAA’s Coastal Flood Exposure Mapper was provided by an NOAA affiliate in the form of one raster file per US state and was limited to only those pixels with a hazard number of 1 or greater (i.e., those pixels at risk to one or more flooding hazards) (Table 1). Shapefiles containing inland flood data, derived from FEMA’s National Flood Hazard Layer, were downloaded from Data.gov and limited to only those areas with some degree of risk. Earthquake data were downloaded as polygon shapefiles from USGS’s Short-Term Induced Seismicity Models site and were limited to only those areas with at least a 1% chance of damage. Despite these hazards’ differences from the six previously mentioned, a definition query, the Dissolve tool, and the Summarize-Within tool were all used to calculate possible exposure.

Traditionally, a series of maps would illustrate data patterns over space and time. Still, within the Space Time Pattern Mining toolbox in ArcGIS Pro several tools are offered to bolster analysis and help simplify the visualization of spatiotemporal data. The “Create Space Time Cube From Defined Locations” tool was used to create a space time cube (STC) for each hazard with 10+ years of percent area values. This analysis could not be performed on coastal and inland flooding or earthquakes due to a lack of temporal data. In this case, defined locations, or the spatial component of the STC, were US counties. The time variable, or the third dimension of the cube, was yearly percent area values associated with each county. Each hazard’s cube was stored as a netCDF data cube and analyzed for trends in percent area at each county. The Emerging Hot Spot Analysis (EHSA) tool and 2D and 3D visualizations were used to further analyze each STC. An EHSA and 2D visualization of the percent area trends map was created for each of the six hazards. For all EHSA trials, the neighborhood time step was defined as one year, and the conceptualization of spatial relationships was defined as contiguity edges only. Refer to the Supplemental Material for more information on the Space Time Pattern Mining toolbox.

In addition to EHSA and 2D visualization maps, the IDW (Inverse Distance Weighted) tool was used to map an interpolated continuous surface of annualized percent area values for each year group for each hazard. Prior to running this tool, annualized or mean percent of county area values were calculated for each hazard for each year group, these being: 2000–2004, 2005–2009, 2010–2014, 2015–2019, and 2000–2019. Once calculated, the shapefile containing this data was converted to a point shapefile for use in the IDW tool. Within this tool, the search radius was defined as a fixed distance with a minimum number of points equal to all points or counties within the US; all other defaults were accepted. Individually, each of the three tools used to create maps offers a unique perspective on possible hazard exposure across the US. Still, when considered together, they provide a much more telling story.

## 3. Results and Discussion

**Figure 2.**
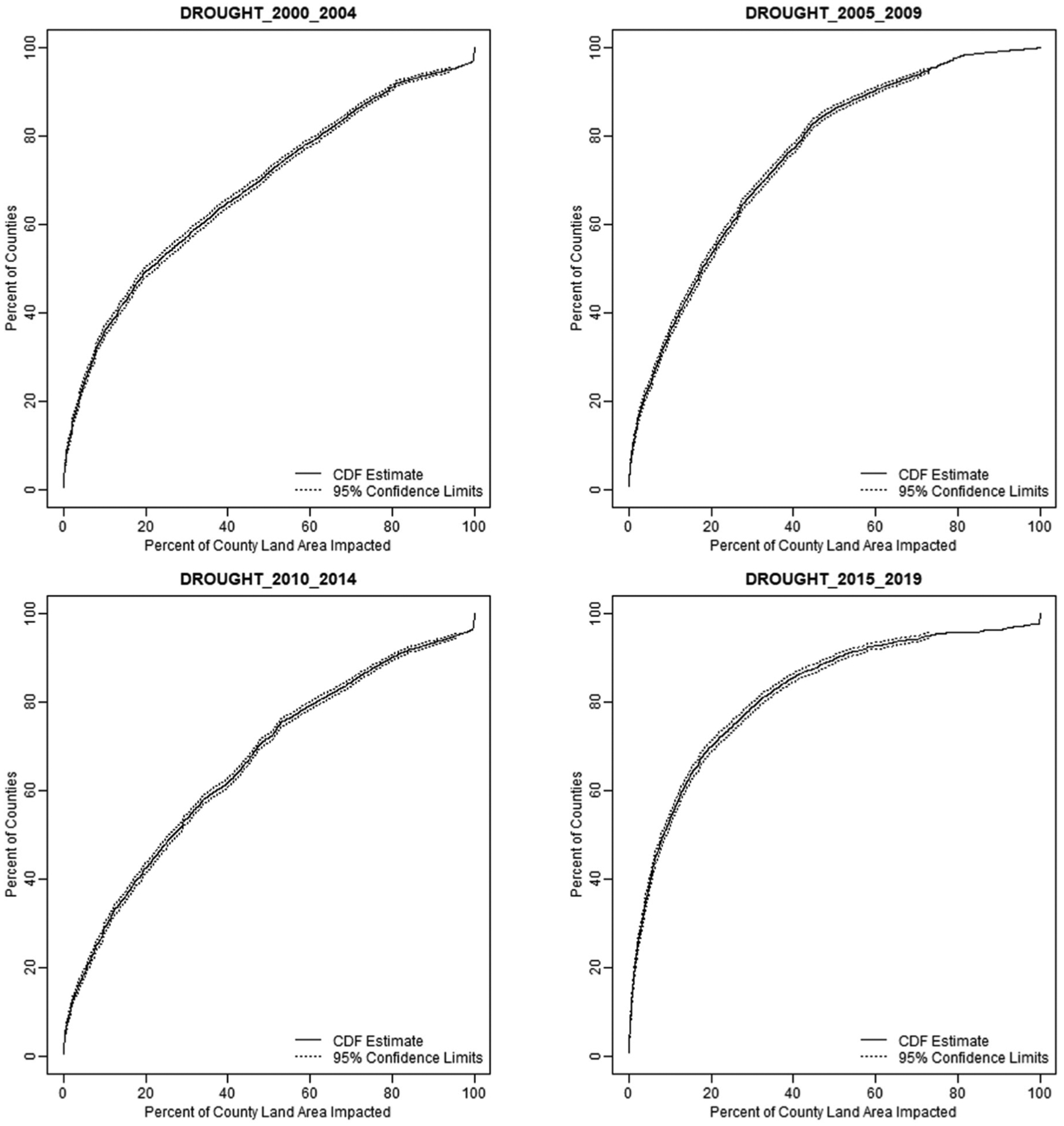
Example of cumulative distribution function (CDF) for drought in 5-year increments (excludes counties with no drought).

Table 2 shows the number of counties assessed for each natural hazard, the number of counties likely impacted and not impacted by each natural hazard, and the proportion of US counties impacted by each hazard in the observed time increments. Inland flooding and drought occurred in a significant number of counties (>30%); inland flooding was observed in 93% of counties. Coastal flooding, earthquakes, tropical storms and hurricanes, tornadoes, and wildfires occurred in a moderate number of counties (~10%), while landslides were observed in less than 1% of counties. Cumulative distribution functions were created only for the counties where the natural hazard was observed during the period 2000–2019. An example of the cumulative distribution frequencies (CDFs) for drought occurrences in U.S. counties from 2000–2019 in five-year increments is shown in Figure 2. The incremental CDFs for drought represent about 1000–1500 counties during 2000–2019. These CDFs depict that cumulatively 20% of counties with drought experience the hazard in about ≤6% of their land area; cumulatively 40% of counties with drought experience it in about ≤18% of their area; cumulatively, 60% of drought counties have drought in about ≤37% of their area; and cumulatively 80% of drought-stricken counties have the hazard in ≤62% of their area. CDFs for the remaining natural hazards are shown in the Supplementary Materials (Figures S1–S8).

A summarization of the time incremental natural hazard CDFs is provided in Table 3 as the inflection points representing quintiles for each natural hazard. Using the Kolmogorov–Smirnov (KS) test (Table 3), significant time differences were observed in the frequencies for hurricanes, tropical storms, and drought. In contrast, no significant differences were observed for tornadoes, landslides, and wildfires throughout the U.S. between 2000 and 2019. This does not mean that there have been no changes in tornadoes, landslides, or wildfires. The observed number of events across the U.S. has not changed significantly with observed changes in overall climate for the 20-year period. Significant changes in intensity and location of the natural hazards are still possible. While the intensity of individual events was not directly measured, an indication of intensity can be seen from the spatial extent of a natural hazard (e.g., how far inland a hurricane tracks and results in damage or the average size of wildfires). KS tests could not be performed for coastal flooding, inland flooding, or earthquakes as the time series data are not available for these hazards.

**Figure 3.**
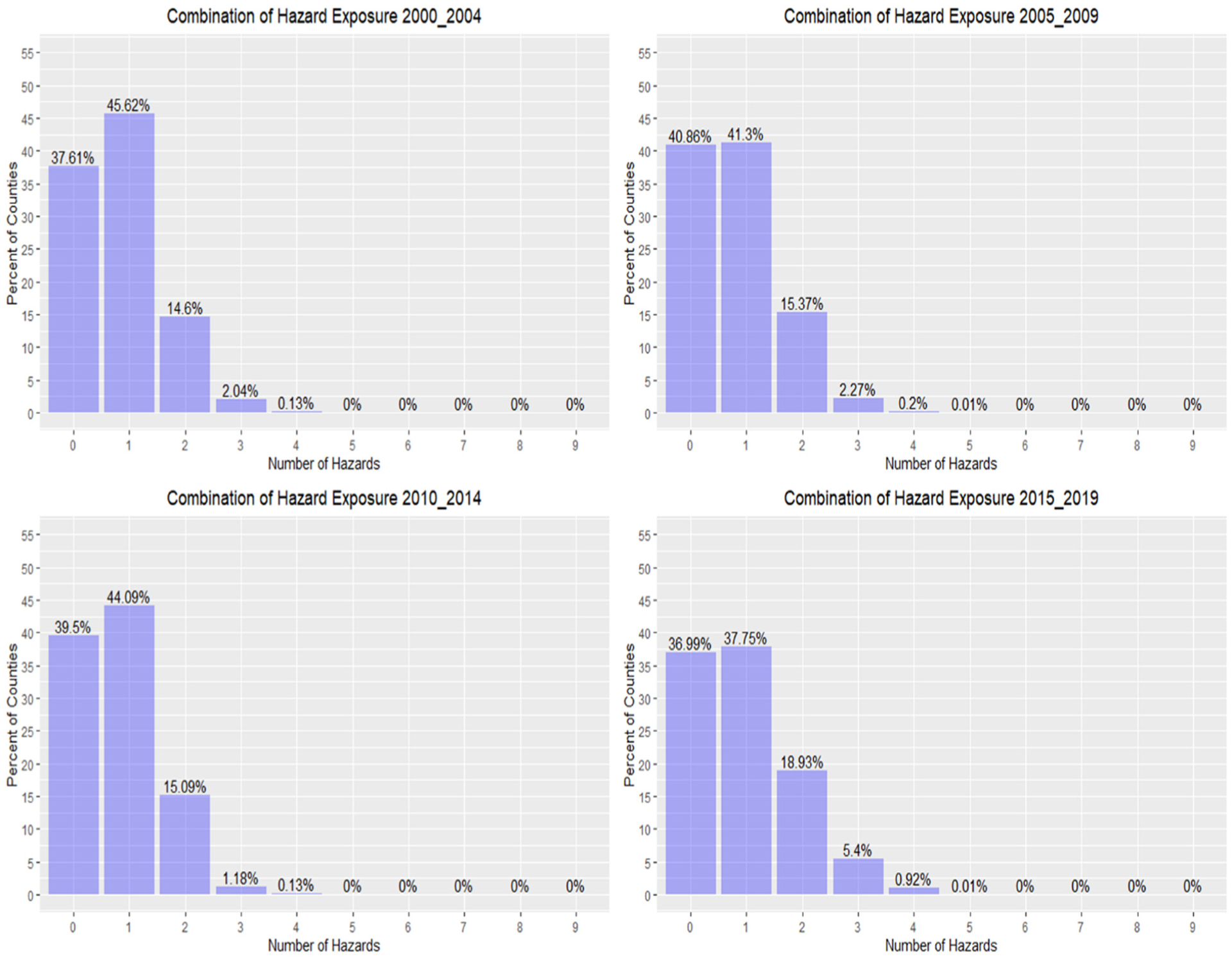
Percentage of counties in US encountering multiple natural hazards during five-year in-crements from 2000–2019.

Another approach for examining frequencies of events is to assess the number of different types of natural hazards that affect a single county during the five-year increments. The number of different natural hazard events experienced by a county during each of the time periods shows a 51% increase in the percent of counties experiencing two or more natural hazards from 2000–2004 (16.8%) to 2015–2019 (25.3%) (Figure 3). Counties experiencing no natural hazards ranged from 37–41%, while those encountering only one hazard ranged from a low of 38% in time period four (2015–2019) to a high of 46% in time period one (2000–2004) (i.e., 18% decrease). The number of counties encountering four or more natural hazards during a time period increased seven-fold.

While the frequency of events increased for only three of the six hazards that could be tested, the remaining three hazards (tornadoes, landslides, and wildfires) are relatively place-specific hazards. This means that hurricanes, tropical storms, and drought are relatively widespread in the areas where they occur (i.e., impact multiple counties). This is also likely for flooding (inland and coastal). Tornadoes, landslides, wildfires, and earthquakes events generally occur in a single county, and, as such, the expectation would be that frequency of impact on the land area might take more than 20 years to depict. Another approach to examining an increasing trend in these area-specific natural hazards would be to examine the specific locations of the hazards (spatial distribution) over the twenty-year period.

**Figure 4.**
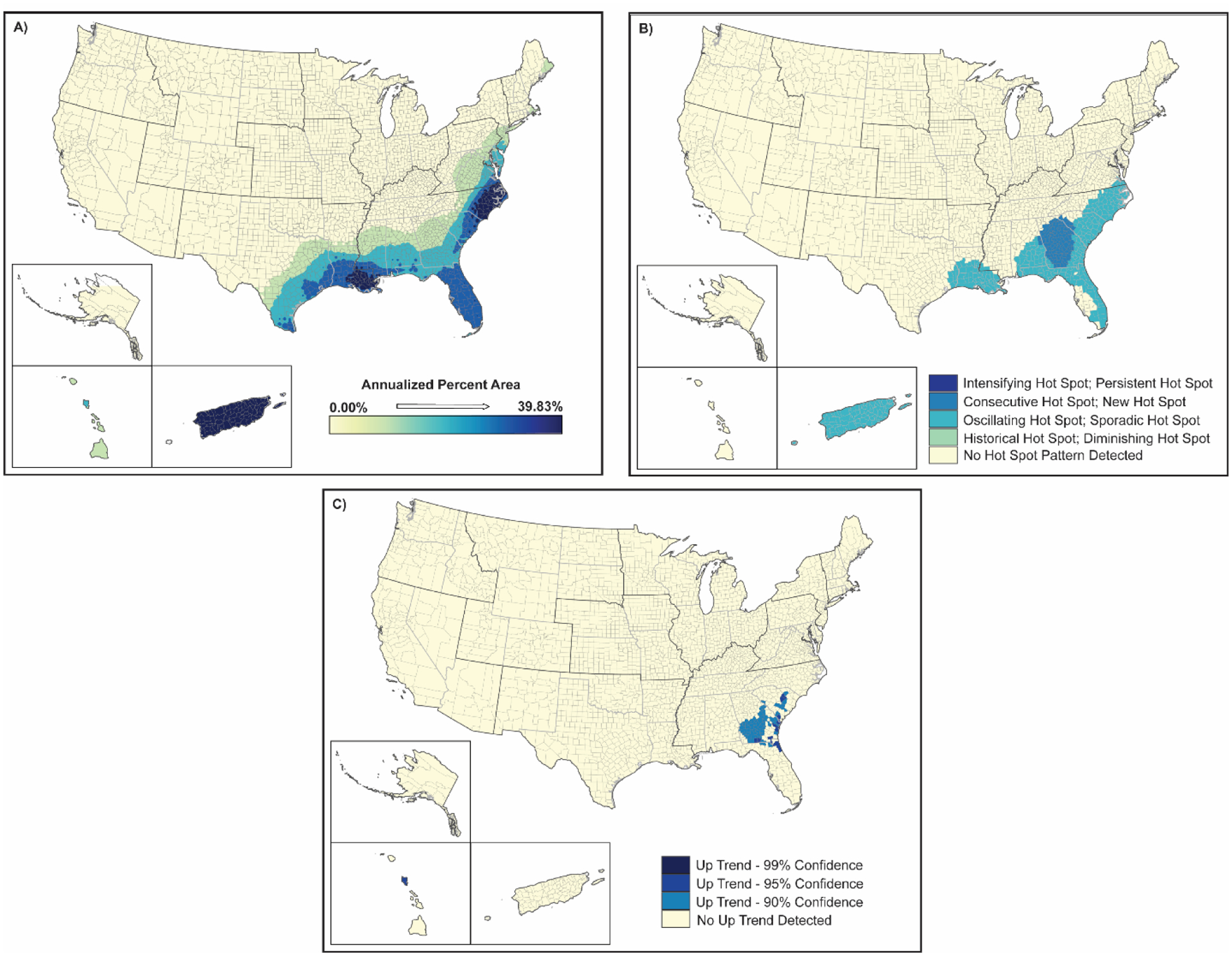
Spatial analysis results for hurricanes (2000–2019) showing (**A**) inverse-distance model of percent area impacts of hurricanes, (**B**) emerging hot spot analysis by hot spot type, and (**C**) counties demonstrating upward trends in percent area by confidence level.

**Figure 5.**
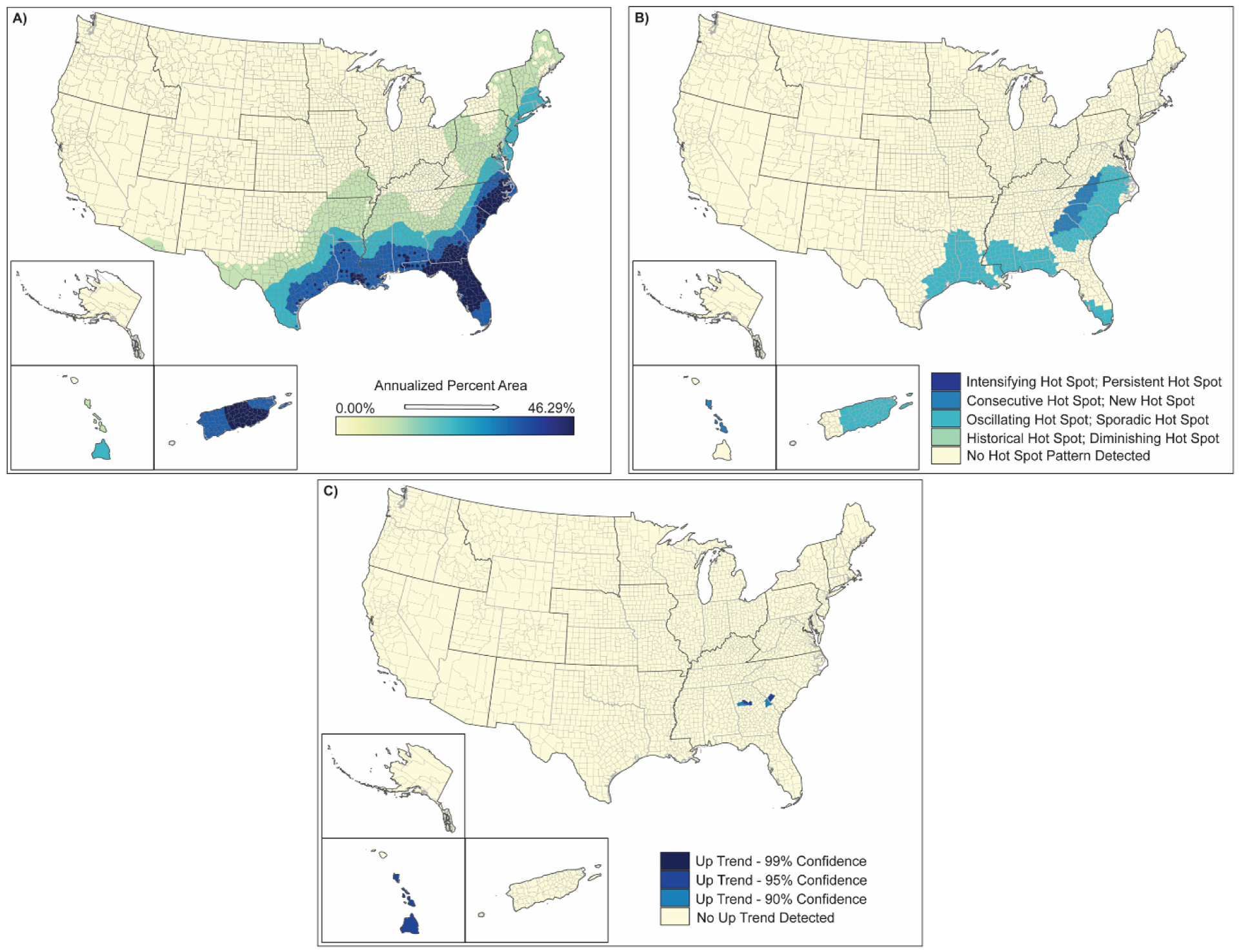
Spatial analysis results for tropical storms (2000–2018) showing (**A**) inverse-distance model of percent area impacts of tropical storms, (**B**) emerging hot spot analysis by hot spot type, and (**C**) counties demonstrating upward trends in percent area by confidence level.

The spatial distributions of hurricanes and tropical storms are shown in Figures 4 and 5. These figures generally illustrate the annualized percentage of the area impacted by these hazards (map a), a spatial analysis of emerging hot spots (map b), and upward trending counties in terms of impacted area for the 2000–2019 time period (map c). While the frequencies of hurricanes and tropical storms were shown to significantly change over the twenty-year period concurrent with changes in climate (Table 3), the spatial distribution of these hazards is targeted in Puerto Rico, most of Florida, southern Georgia, coastal Louisiana, and the Carolina coasts (Figures 4A and 5A) with generally more than 30% of the landmass in counties in these areas being exposed to hurricanes and tropical storms. Similarly, developing spatio-temporal hot spots for hurricanes were observed throughout the southeastern Atlantic coast, coastal Louisiana, Puerto Rico, and areas in south and central Georgia showed some hot spot development (Figure 3). Tropical storms showed a similar but more pervasive hot spot development spreading westward through much of the Carolinas and northward into central portions of Alabama, Mississippi, and Louisiana as well as inland areas of Texas (Figure 5B). Upward trending counties for both hurricanes and tropical storms (Figures 4C and 5C) mirrored the encroachment of these natural hazards deeper into the interior of southern and southeastern states. These trends also signify likely increases in intensities of these hazards over the twenty-year time span. These increases in frequency and intensity (i.e., expanded extent) of hurricanes and tropical storms coincide with increasing evidence of climate change (e.g., increasing temperatures, changing wind patterns, changing ocean temperature). Additional spatial information for hurricanes and tropical storms is provided in the Supplemental Materials (Figures S9–S12).

The data reported here support the inferences connecting increased frequency and intensity of hurricanes and tropical storms to climate change [37–39]. Similarly, the extended datasets for hurricanes and tropical storms, as well as the frequency tests, spatial distribution analyses, and intensity evaluations, support previous modeling efforts [40] and spatial analysis projections [41]. Among the nine natural hazards examined, hurricanes and tropical storms appear to have the strongest associations with climate change. Although outside the scope of the datasets developed here, the hurricanes/tropical storms of 2020 and 2021 further support the hypothesis that increases in storm frequency, intensity, and concentration of spatial placement are concurrent with climate changes. The U.S. normally averages about three named storms per year, but the U.S. had 11 named storms in 2020 and 21 named storms in 2021. Hurricane/Tropical Storm/ Rain Event Ida lasted almost two weeks (23 August 2021 through 4 September 2021) and created hurricane destruction from south-central coastal Louisiana to New York City, causing major flooding.

**Figure 6.**
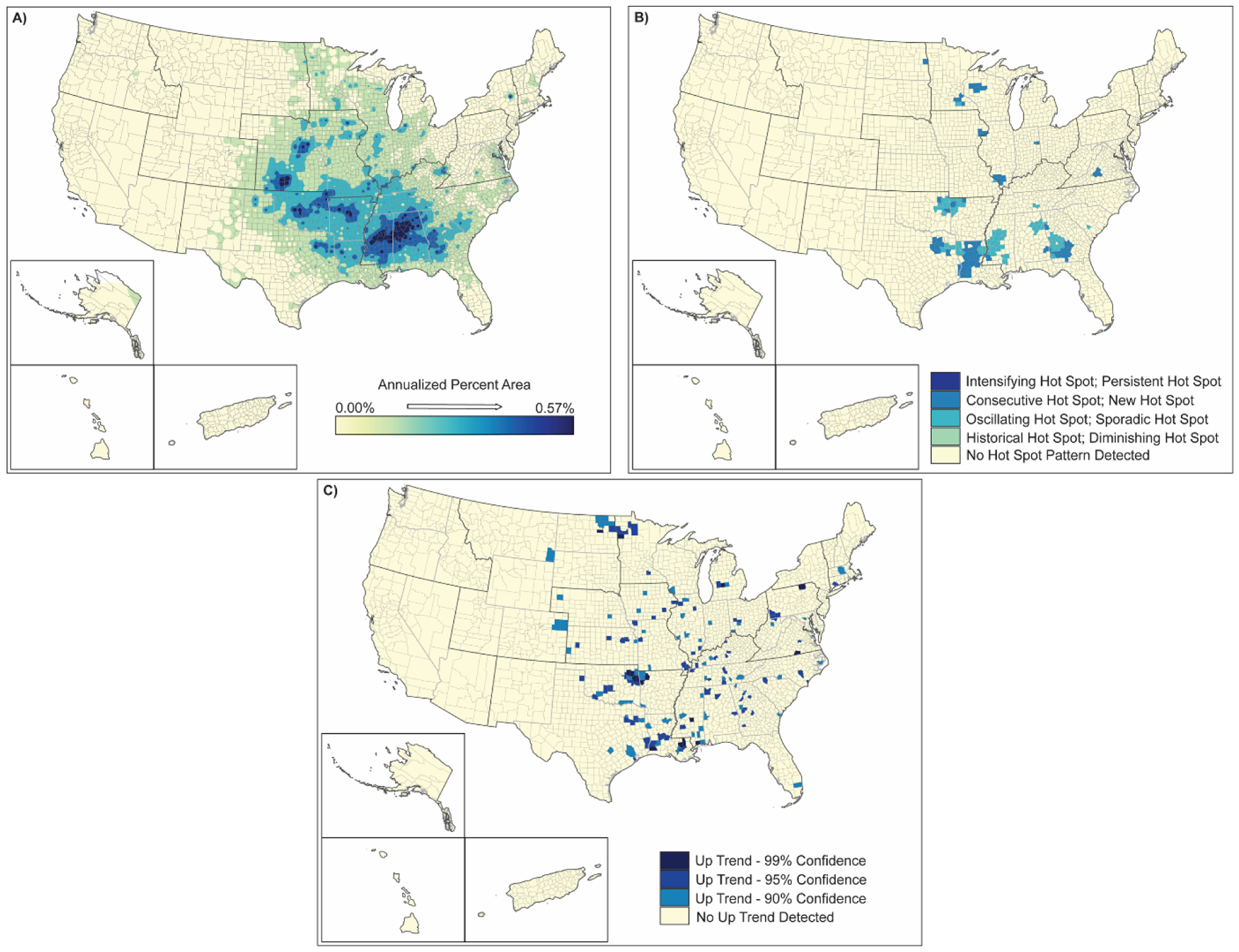
Spatial analysis results for tornadoes (2000–2019) showing (**A**) inverse-distance model of percent area impacts of tornadoes, (**B**) emerging hot spot analysis by hot spot type, and (**C**) counties demonstrating upward trends in percent area by confidence level.

Tornadoes affect 13–16% of the counties in the US (Table 2) and <9% of the land area in those counties (Table 3). Tornado frequency does not appear to have changed significantly over the 2000–2019 period (Table 3); the areas most affected are located in the southeast, south, and central plains (Figure 6A). The areas of strongest impact are located in a belt stretching from southwest Georgia to southwestern Iowa, with the highest areal impacts in Iowa, Oklahoma, Arkansas, Mississippi, and Alabama. Other isolated areas are distributed throughout the central US and areas to the east. Hot spot areas for tornadoes focus in areas away from “Tornado Alley” to the south in central and northern Louisiana, northeastern Oklahoma, and northwestern Arkansas, and southwestern Georgia (Figure 6B). These hot spot regions suggest a spreading or widening of the traditional tornado zone. The counties trending upward are spread throughout the country from Montana-Colorado to the east coast but in small distinct areas that are typical of tornado occurrence (Figure 6C). Although the increased frequency of tornadoes is not significant, the widespread spatial increase in localized up-trending areas throughout the eastern half of the US suggests an increase in the extent of this hazard coincident with the changing climate over the past twenty years.

Climate history extensions of tornado frequency and intensity, as well as spatial hot spot analyses and trend assessments, support earlier conceptual linking of severe thunderstorm and air circulation patterns resulting in tornadoes [14]. The analyses reported here similarly support utilizing synoptic climatological methods to assess the impacts of climate change on future tornado-favorable environments [16]. While increased variability in tornado occurrences has been documented [42], their connection to climate change remains unresolved. Our spatial analyses suggest the possibility of a connection but, because of the site-specific nature of the hazard, likely cannot be confirmed without a long record of data [43] Similar to hurricanes and tropical storms, characteristics in tornadoes changed in 2021 to larger storms, storms occurring later in the year, and storms that tracked for greater distances. In December 2021, multiple tornadoes (at least 50) occurred in a 12 h period in the central Mississippi basin (i.e., along the Arkansas, Illinois, Indiana, Kentucky, Mississippi, Ohio, and Tennessee corridor). Some of these storms tracked over 125 miles; thus, being very different types of tornadoes than the site-specific storms dominant in the 2000–2019 period. These storms resulted in over 100 deaths and billions of dollars in property damages. One of these tornadoes stretched for more than 250 miles from Arkansas to Kentucky (https://www.spc.noaa.gov/exper/reports/?&all&date=20211210; https://www.cnn.com/2021/12/11/weather/severe-weather-tornadoes-saturday/index.html) (accessed on 15 January 2022).

**Figure 7.**
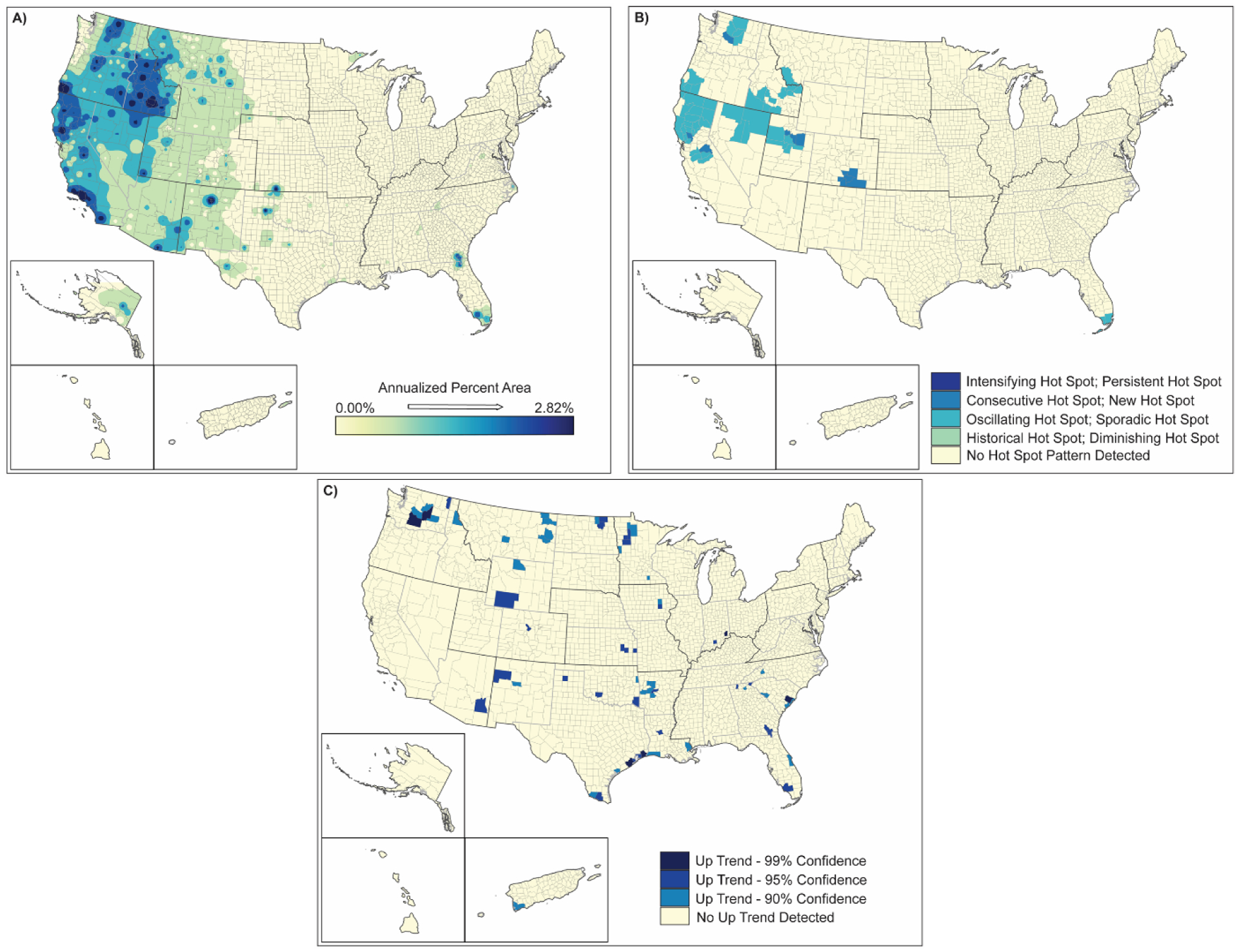
Spatial analysis results for wildfires (2000–2019) showing (**A**) inverse-distance model of percent area impacts of wildfires, (**B**) emerging hot spot analysis by hot spot type, and (**C**) counties demonstrating upward trends in percent area by confidence level.

As expected, wildfires are largely located in the western half of the US. While wildfires are broadly distributed, affecting only 9–10% of US counties (e.g., due to the large size of western counties) (Table 2), they impact 1–64% of the areas in those counties (Table 3). Similar to tornadoes, individual wildfires tend to be county-specific. With the large size of western counties where wildfires occur, the annualized percent area of counties affected is higher than that of tornadoes (Figure 7A). However, the area in most counties that are affected is small. Hot spot analyses show that wildfires are intensifying in northern California, northern Nevada, some areas of Utah, Idaho, northern Washington, and southern Colorado (Figure 7B). Strong upward trends in wildfire occurrences and intensity appear to exist in select counties in south-central Washington, Arizona, New Mexico, Wyoming, North Dakota, southwestern Puerto Rico, and the northeastern Texas coast (Figure 7C).

Like tornadoes, the spatial distribution of an individual wildfire is very site-specific, rarely occurring outside a single county. This distribution makes it difficult to confirm increasing frequencies, intensities, or spatial distributions of wildfires without significantly more temporal data. However, even with these limitations, the extended wildfire histories appear to confirm projections described by others with regard to modeled relationships to climate change [44]. Wildfires appear to be spreading to “new” areas (northern Washington), and the size and intensity of wildfires appear to be increasing based on our spatial analyses and confirms projections by others [45].

**Figure 8.**
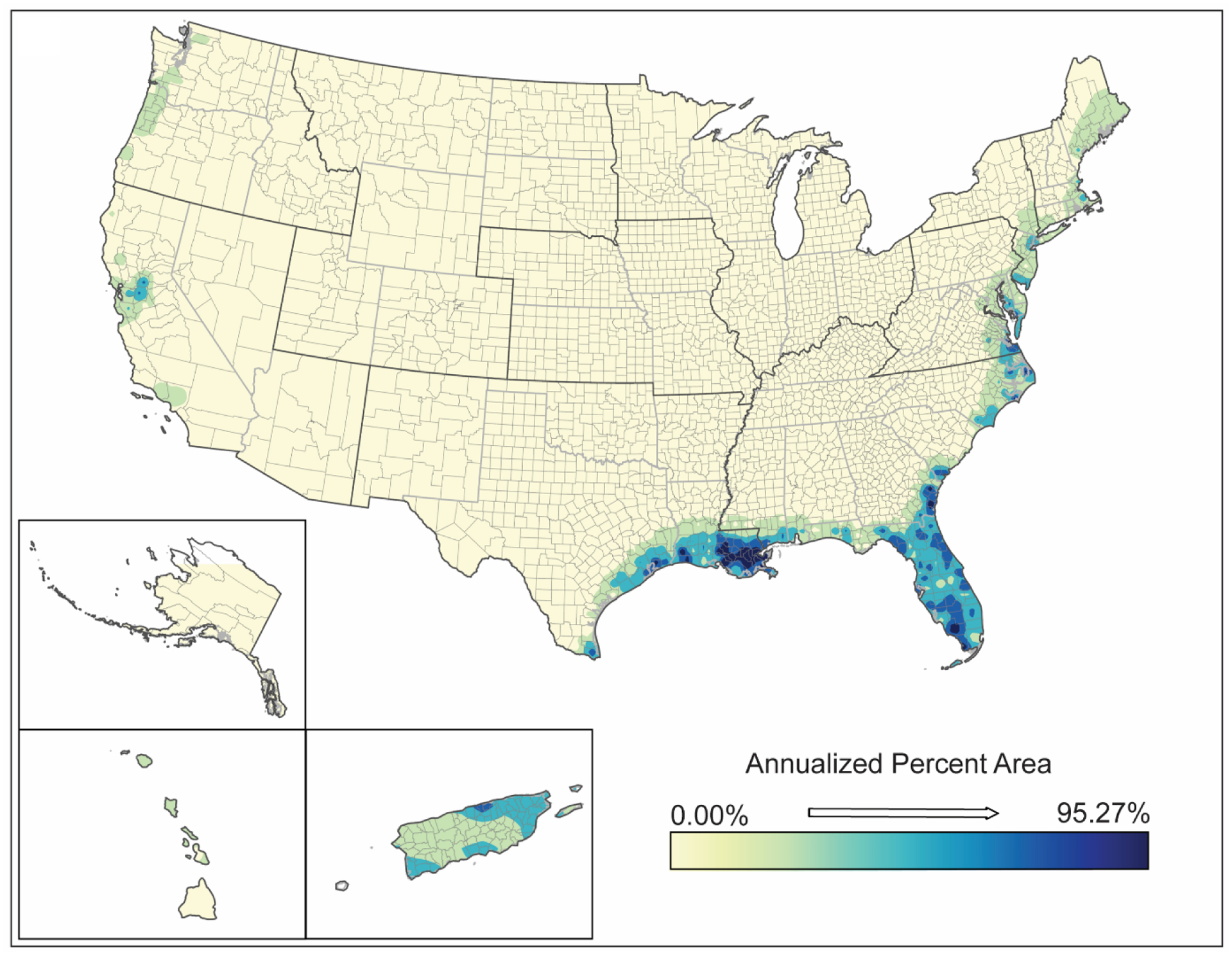
Spatial analysis results for coastal flooding (2019) showing inverse-distance model of percent area impacts of coastal flooding.

**Figure 9.**
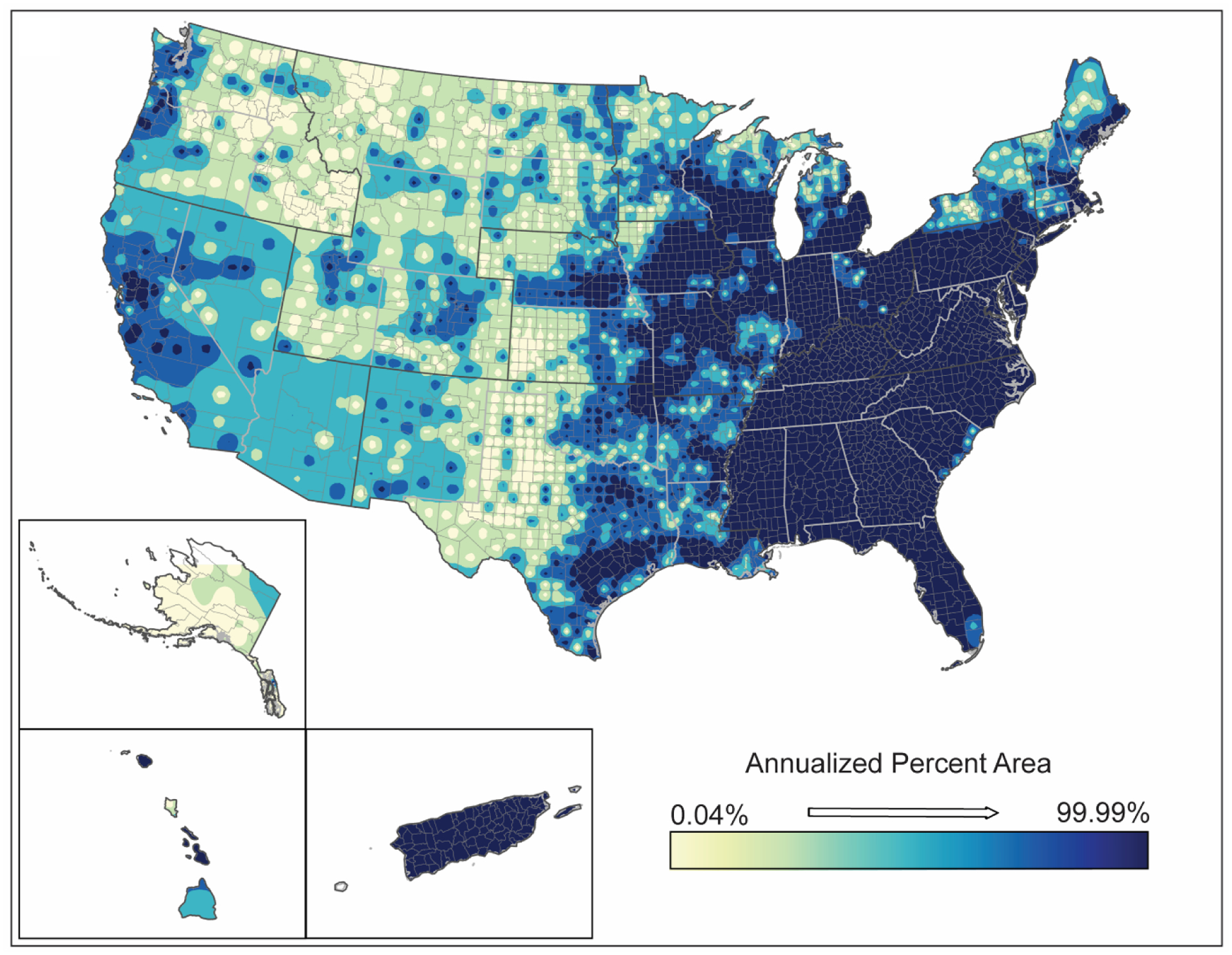
Spatial analysis results for inland flooding (2019) showing inverse-distance model of percent area impacts of inland flooding.

No hot spot or trend analyses could be completed for flooding (inland or coastal) or earthquakes due to data only being available for the period 2015–2019. Even with a relative paucity of information (compared to other natural hazards), coastal flooding occurs in about 11% of US counties (345 counties) which corresponds to nearly all coastal counties along the east coast and Gulf of Mexico coast. About 60% of those counties are impacted across 100% of their land areas (Tables 2 and 3). Similarly, 2998 counties (93% of US counties) are most likely exposed to some inland flooding impacting <26% of the area for 20% of the counties. (Tables 2 and 3). Coastal flooding is particularly impactful in areas of south Louisiana, southern Florida, northeastern coastal Texas, and Puerto Rico (Figure 8). Unlike coastal flooding, which is restricted to eastern and southern coastlines, inland flooding, which is restricted to eastern and southern coastlines, inland flooding most likely heavily impacts almost all of the United States east of the Mississippi River and all of Puerto Rico. Additional areas heavily impacted include southern Minnesota, southern Michigan, eastern Iowa, northern Missouri, south-central Texas, southwestern Louisiana, and selected Hawaiian Islands (Figure 9).

Hypothesized relationships between flooding (both coastal and inland) and climate change are numerous [46–49]. While our datasets cannot be used to support these conjectures, the data from 2015–2019 suggest the possibility of widespread occurrences of inland flooding (Figure 9), which seems to represent the alternate image of the spatial distribution of drought where inland flooding occurs east of the Mississippi River and drought occurs primarily to the west.

**Figure 10.**
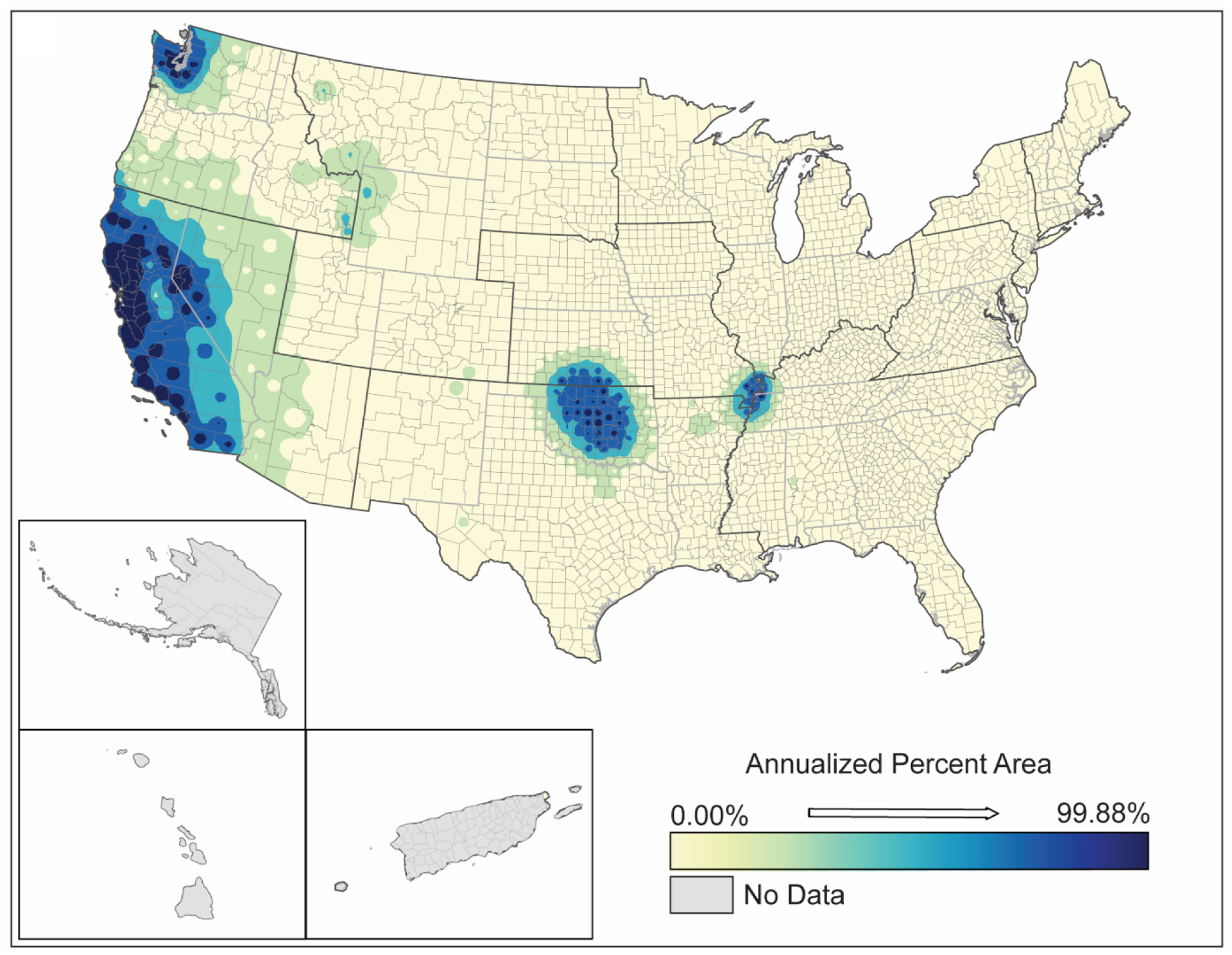
Spatial analysis results for earthquakes (2019) showing inverse-distance model of per-cent area impacts of earthquakes.

With similar data availability, analyses of annualized percentage of the area impacted by earthquakes were restricted to the 2015–2019 time period. Earthquakes were observed in 681 counties (7%), with impacted areas in 60% of these counties of <99% of their land areas (Tables 2 and 3). Earthquakes are concentrated primarily in four areas of the US—California and western Nevada, the Puget Sound region of Washington, central Oklahoma and south-central Kansas, and the four corners area of Missouri/Kentucky/Tennessee/Arkansas (Figure 10). Lower impacted areas exist throughout western Arizona, eastern Nevada, southern Oregon, eastern Idaho, southwestern Montana, and western Wyoming.

Oliver-Smith and Hoffman [50] have suggested a potential relationship between earthquake occurrence and climate change; however, our dataset does not provide any support for this hypothesis. Earthquakes are primarily tectonic in nature. Natural earthquakes are caused by fault movements, although some human-induced earthquakes can be caused by explosions, fracking, and water injection. Climate change “might” affect this latter category of earthquakes by evidence is limited thus far. The best support the present dataset provides is that earthquakes appear to be concentrating in central Oklahoma and the western United States (i.e., California and western Nevada). The increasing number of earthquakes in Oklahoma has been attributed to human-induced climate change or a deep well injection of coproduced water from oil and gas wells [51].

**Figure 11.**
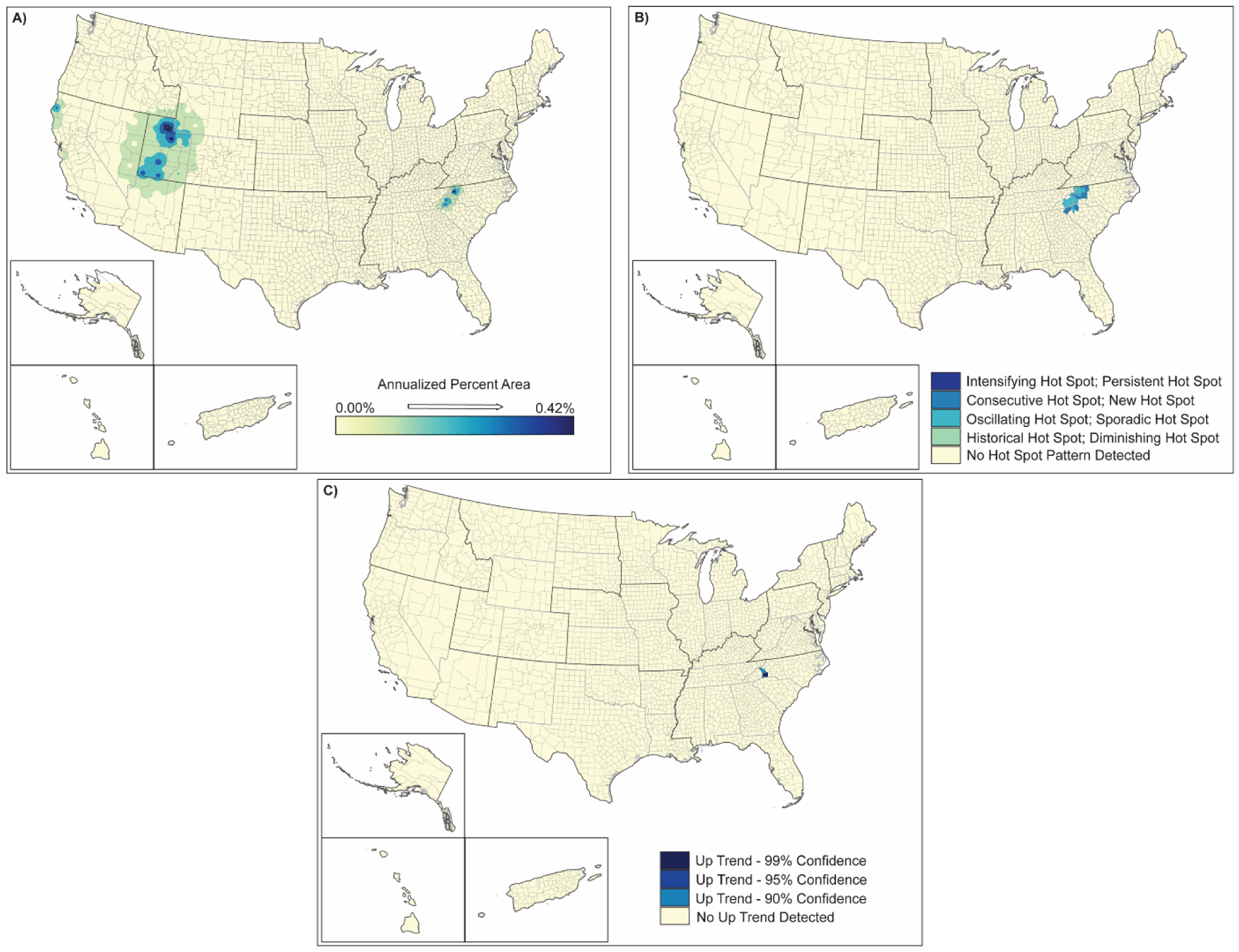
Spatial analysis results for landslides (2000–2018) showing (**A**) inverse-distance model of percent area impacts of landslides, (**B**) emerging hot spot analysis by hot spot type, and (**C**) counties demonstrating upward trends in percent area by confidence level.

Landslides, similar to tornadoes and wildfires, are site-specific and occur in <1% of US counties and impact 1–7% of the area in those counties (Tables 2 and 3). Spatial analyses of the annualized percent area of impact show strong impacts (<0.5% of land area) in some counties in north-central Utah and moderate impacts (<0.2% of land area) in most of the remaining counties in Utah and in North Carolina/Tennessee Appalachian Mountains (Figure 11A). Hot spot and upward trend analysis were restricted to counties in the North Carolina/Tennessee Appalachian Mountains (Figure 11B,C).

Landslides tend to be a higher-order effect of climate change. Hence, relations between climate change and landslide response are highly diverse in type, space, and time. While landslides are generally site-specific, the enhanced landslide histories depicted for the period 2015–2019 seem to support the hypotheses that increase in the number of landslides, size of landslides, and locations of landslides are related to climate change or the results of climate change (e.g., increases in precipitation, increases in earthquakes) [52,53].

**Figure 12.**
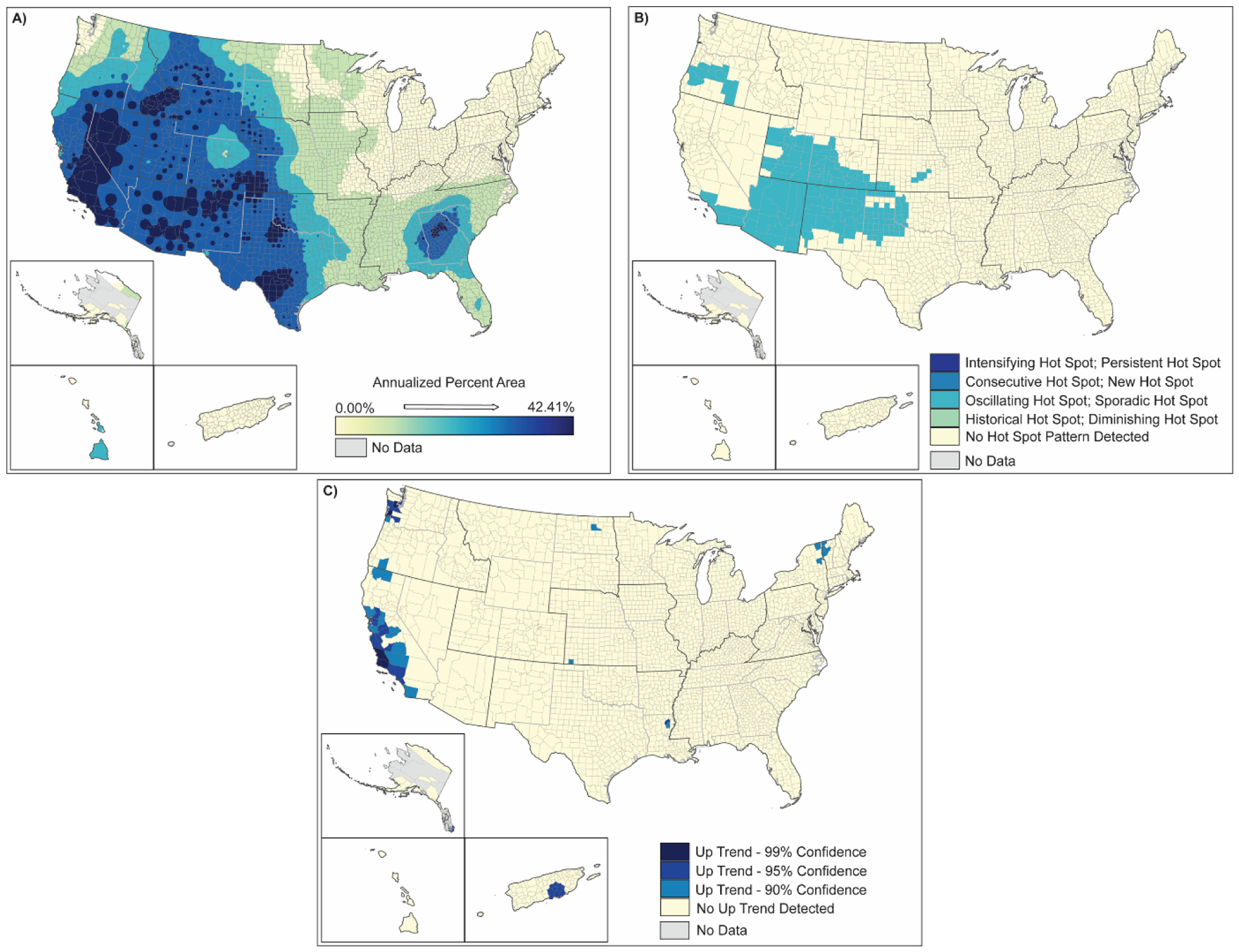
Spatial analysis results for drought (2000–2019) showing (**A**) inverse-distance model of percent area impacts of drought, (**B**) emerging hot spot analysis by hot spot type, and (**C**) counties demonstrating upward trends in percent area by confidence level.

The occurrence of moderate to severe drought (a proxy for lack of rainfall/snowfall) occurred in 30–45% of US counties during 2000–2019, impacting >80% of the land area of 40% of these counties (Tables 2 and 3). Spatial analyses of the drought data show that almost all of the US except for the Northeast and North-Central US was significantly impacted by drought (Figure 12A). The areas of greatest impact occurred west of the Mississippi River, especially south and central California, western Nevada, and specific counties throughout the southwest and central-western US. The Hawaiian Islands of Hawaii and Maui were also significantly impacted. Hot spot analyses show that while central California and northern Nevada were areas of significant drought in 2000–2019, areas more to the south and east of these areas were the new hot spot areas for drought. These include most of Arizona, Utah and New Mexico, southern California, western Colorado, northern Texas, and a band of counties through central Oregon (Figure 12B). While these county areas showed up as significant hot spots in 2000–2019, the up-trending counties in the US were along the California coastline from San Diego to San Francisco, central California, south-central Puerto Rico, north-central California, and the coastal counties of northern Washington (Figure 12C).

One of the more straightforward relationships in the enhanced natural hazard histories is the connection between drought and climate change. The data support hypothesized increases in drought severity [54], the extent of moderate and extreme drought [55], drought risk [56], and higher-order impacts resulting in wildfires [57].

## 4. Conclusions

The primary goal of this data compilation and analysis was to determine if objective evidence could be determined related to changes in the frequency, intensity, severity, and spatial location of nine natural hazards. The secondary goal was to determine if these potential changes supported inferences concerning the changes and climate change. For drought, hurricanes, and tropical storms, there was clear evidence of these increases in frequency and intensity and changes in the spatial location of the events. For inland flooding, coastal flooding, and earthquakes, there was some support for climate change hypotheses, but strong data were unavailable for the entire period preventing statistical testing. The remaining hazard events (tornadoes, landslides, and wildfires) also appeared to support existing inferences of their potential relationships to climate change, but due to the site-specific nature of these events, it is unlikely that statistically significant changes will be observed without a longer time series of data. However, even this last group of natural hazards showed strong changes in the development of spatial hotspots and spatial trends in location. Similar to others, we believe that the majority of these natural hazards will be altered by ongoing climate change. In addition, we contend that the development of these datasets and the CDF analyses and spatial analyses for the period 2000–2019 provide some objective evidence that these perceived changes are real and that, without strong anthropogenic responses to climate change, these natural hazards will continue to worsen.

Analyses showed clear changes in the frequency and intensity of hurricanes, tropical storms, and drought that can be related to climate change factors. Internal and coastal flooding also demonstrated these connections, although the length of the dataset did not permit significant testing but shows significant hot spots and trending locations. Tornadoes, landslides, and wildfires showed significant hot spots and trending locations, but due to the specific locational nature of the data, they did not show significant changes in frequency. Earthquakes showed no significant changes over the time period.

## Supplementary Material

Supplement1

## Figures and Tables

**Table 1.** Natural hazard secondary data source, temporal extent, and spatial extent information (CONUS = contiguous United States). (The accessed dates for the links are 15 January 2022).

Natural Hazard Type	Secondary Data Source	Temporal Extent (Target Years: 2000–2019)	Spatial Extent (Target Regions: CONUS, AK, HI, and PR)
*Hurricanes and Tropical Storms*	International Best Track Archive for Climate Stewardship (IBTrACS) https://www.ncdc.noaa.gov/ibtracs/index.php?name=ib-v4-access	1980–2021	CONUS, AK, HI, and PR
*Tornadoes*	NOAA’s Storm Prediction Center (SPC) https://www.spc.noaa.gov/gis/svrgis/	1950–2018	
*Landslides*	USGS’s Landslide Inventories across the United States https://www.sciencebase.gov/catalog/item/5c7065b4e4b0fe48cb43fbd7	1900–2018	
*Wildfires*	National Interagency Fire Center, Interagency Fire Perimeter History —All Years https://data-nifc.opendata.arcgis.com/datasets/interagency-fire-perimeter-history-all-years/explore?location=43.578757%2C63.134208%2C3.57	1835–2019	
*Drought*	US Drought Monitor https://droughtmonitor.unl.edu/DmData/DataDownload.aspx	2000–2020	CONUS, *Most* of AK, HI, and PR
*Coastal Flooding*	Coastal Flood Exposure Mapper, Coastal Flood Hazard Composite Layer https://coast.noaa.gov/floodexposure/#-10575352,4439107,5z	2019	CONUS, HI, and PR
*Inland Flooding*	National Flood Hazard Layer (NFHL), Seamless Nationwide NFHL GIS data https://catalog.data.gov/dataset/national-flood-hazard-layer-nfhl	2019	CONUS, HI, AK, and PR
*Earthquakes*	Short-term Induced Seismicity Models https://www.usgs.gov/natural-hazards/earthquake-hazards/science/short-term-induced-seismicity-models?qt-science_center_objects=0#23qt-science_center_objects	2016–2018	CONUS

**Table 2.** The proportion of counties not exposed to natural hazards in five-year increments.

Natural Hazard	Year Increment	Number of County-Year Combinations	Counties Not Impacted	Counties Impacted	Percent Counties Impacted
Coastal Flooding	2015–2019	3220	2875	345	10.71
Inland Flooding	2015–2019	3220	222	2998	93.11
Earthquakes	2015–2019	9324	8643	681	7.30
Drought	2000–2004	16,040	8845	7195	44.86
	2005–2009	16,040	9684	6356	39.63
	2010–2014	16,040	9110	6930	43.20
	2015–2019	16,040	10,598	5442	33.93
Hurricanes	2000–2004	16,100	15,268	832	5.17
	2005–2009	16,100	15,389	711	4.42
	2010–2014	16,100	15,503	597	3.71
	2015–2019	16,100	15,006	1094	6.80
Tropical Storms	2000–2004	16,100	14,547	1553	9.65
	2005–2009	16,100	14,596	1504	9.34
	2010–2014	16,100	15,314	786	4.88
	2015–2019	16,100	14,767	1333	8.28
Tornadoes	2000–2004	16,100	14,082	2018	12.53
	2005–2009	16,100	13,698	2402	14.92
	2010–2014	16,100	13,451	2649	16.45
	2015–2019	16,100	10,751	2129	13.22
Landslides	2000–2004	16,100	16,029	71	0.44
	2005–2009	16,100	16,052	48	0.30
	2010–2014	16,100	16,086	14	0.09
	2015–2019	12,880	12,861	19	0.12
Wildfires	2000–2004	16,100	14,673	1427	8.86
	2005–2009	16,100	14,533	1567	9.73
	2010–2014	16,100	14,433	1667	10.35
	2015–2019	16,100	14,595	1505	9.35

**Table 3.** Approximation of cumulative distribution functions (Quintiles from 0–100% of counties where data represents percentage of land area in counties).

Natural Hazard	Time Interval	KS Test Interval	0%	20%	40%	60%	80%	100%	KS	AD
Coastal Flooding	4	n.a.	0.01	3.49	11.18	23.45	44.61	100	n.a.	n.a.
Inland Flooding	4	n.a.	0.01	25.86	99.95	99.98	100	100	n.a.	n.a.
Earthquakes	4	n.a.	0.01	15.88	56.43	98.78	100	100	n.a.	n.a.
Drought	1	1 to 2	0.01	3.77	11.80	30.63	60.55	100	***	***
	2	2 to 3	0.01	3.60	12.91	25.17	44.23	100	***	***
	3	3 to 4	0.01	6.42	18.28	37.09	61.65	100	***	***
	4	1 to 4	0.01	1.70	5.17	11.21	25.13	100	***	***
Hurricanes	1	1 to 2	0.01	81.51	100	100	100	100	**	***
	2	2 to 3	0.08	66.38	100	100	100	100	***	***
	3	3 to 4	0.01	79.12	100	100	100	100	***	***
	4	1 to 4	0.02	84.69	100	100	100	100	***	***
Tropical Storms	1	1 to 2	0.01	61.02	100	100	100	100	*	***
	2	2 to 3	0.01	49.95	100	100	100	100	*	***
	3	3 to 4	0.01	56.40	100	100	100	100	***	***
	4	1 to 4	0.01	60.77	100	100	100	100	*	***
Tornadoes	1	1 to 2	0.01	0.01	0.03	0.08	0.22	9.08	n.s.	***
	2	2 to 3	0.01	0.01	0.03	0.09	0.28	7.91	n.s.	***
	3	3 to 4	0.01	0.02	0.04	0.11	0.38	8.80	n.s.	***
	4	1 to 4	0.01	0.01	0.04	0.09	0.29	9.05	n.s.	***
Landslides	1	1 to 2	0.01	0.01	0.07	0.18	0.65	6.07	n.s.	**
	2	2 to 3	0.01	0.04	0.11	0.52	1.31	6.26	n.s.	*
	3	3 to 4	0.01	0.01	0.01	0.01	0.02	0.94	n.s.	n.a.
	4	1 to 4	0.01	0.01	0.03	0.46	1.26	7.11	n.s.	n.s.
Wildfires	1	1 to 2	0.01	0.02	0.05	0.15	0.57	49.27	n.s.	***
	2	2 to 3	0.01	0.02	0.07	0.18	0.64	40.58	n.s.	***
	3	3 to 4	0.01	0.02	00.06	0.17	0.66	33.01	n.s.	***
	4	1 to 4	0.01	0.02	0.05	0.17	0.75	64.05	n.s.	***

Results of cumulation distribution function significance testing using Kolmogorov–Smirnov and Darling tests. (Interval: 1 = 2000–2004; 2 = 2005–2009; 3 = 2010–2014; 4 = 2015–2019) (KS = Kolmogorov–Smirnoff Test and AD = Anderson–Darling significance levels where * = <0.05; ** = < 0.01 and *** = < 0.001) (n.a. = cannot use KS or AD due to data limitation where no data are available for next 5-yr increment; n.s. = not significant).

## Data Availability

Data is available from the corresponding author at summers.kevin@epa.gov.
