# Supplementary material for: Observed Changes in the Frequency, Intensity, and Spatial Patterns of Nine Natural Hazards in the United States from 2000 to 2019
Source: Sustainability. Author manuscript; Available in PMC 2023 Mar 31. (PMC9461684; doi:10.3390/su14074158)

Supplemental Materials

Additional Methods:

There are 3 tools that can be used to create a space time cube, these are create Space Time Cube by aggregating points, create Space Time Cube from defined locations, and create Space Time Cube from Multidimensional Raster Layer. We chose to use the second tool which recognizes defined locations because annual percent area values were calculated for each county feature. All 3 tools use the Mann-Kendall statistic to measure trend, in this case percent area values, over time at each location or county. The trend for each county over time is stored as a z-score and a p-value. STC results are stored in a netCDF data cube. The visualize space time cube in 2D tool was used to visualize trend of percent area values over the 20-year period within each county. In contrast to the EHSA tool, this tool does not determine trend within the context of a neighborhood, meaning each county is analyzed in a vacuum. Further investigation of these trends was warranted.

The EHSA tool was used to succinctly visualize such trends within each hazard's STC. We felt that this tool was vital to our research because of its ability to easily analyze and concisely illustrate 20 years' worth of data across 3,220 total counties. As mentioned previously, the neighborhood for each hazard was defined as 1 time-step interval or year and only those counties that share an edge with the county in question. A single time step was chosen because it was the default, and contiguity edges only was used as the spatial constraint because it seemed like the most appropriate option for locations of various sizes. Considering the size difference of counties in the eastern

US to those in the western US, it wouldn't have made sense to conceptualize a neighborhood by a fixed distance or a one-size-fits-all number of K nearest neighbors. Contiguity edges corners could've been used, but contiguity edges only seemed like the more straightforward option out of the two. Upon completion of the EHSA tool, each county is distilled down to or symbolized by 1 of 17 categories relating to the overall trend found at that specific location. Some categories include new, consecutive, intensifying, persistent, diminishing, sporadic, and so on. These categories can either relate to a hot spot or cold spot, and no trend is also a possibility. Aside from initial trend results generated from executing the space time cube tool, 7 additional analysis variables are added to the accompanying STC once the EHSA tool is run.

To produce these additional variables, 3 core analyses are performed. The first being an analysis of intensity of high and low value clustering, where each county within a given year is analyzed in relation to neighboring counties within that same year or time slice. In terms of the second and third analyses, the Mann-Kendall statistic is used to assess both the time series of z-scores and values at those counties analyzed. Unlike the latter two analyses, the first analysis deals exclusively with z-scores and p-values relative to the Getis-Ord Gi* statistic. The Getis-Ord GI* statistic determines if a county is a significant hot or cold spot. For example, a county with a high percent area value within a given year surrounded by neighboring counties with high percent area values would be considered significant and labeled as a hot spot. For more information on the EHSA tool and the 3 core analyses mentioned here, please visit https://pro.arcgis.com/en/pro-app/latest/tool-reference/space-time-pattern-mining/emerginghotspots.htm.

As mentioned previously, the IDW tool was yet another tool used to visualize the spatial distribution of these hazards over time. This tool does not analyze statistical significance or trends. It simply provides an interpolated surface of one set of values. The IDW tool assumes that influence decreases with distance or, in this case, a county centroid further away from the location in question would have less influence over the interpolated value than a closer one. As mentioned previously, the search radius was defined as a fixed distance with a minimum number of points equal to all points or counties within the US (3220 total counties). This was done so that every county centroid would be included within the search radius and used for interpolation. We felt that setting an arbitrary value for either a variable or fixed search radius would contradict our purpose for using this tool, which was to show possible hazard extent without being constrained by political boundaries. In addition to illustrating possible extent via a continuous raster surface, IDW maps, specifically those within the supplemental materials section, help to shed some light on each hazard's EHSA map. For example, a collection of counties considered to be consecutive hot spots for a given hazard would also be shown as having possibly experienced some degree of exposure within multiple year groups or IDW maps. For more information on the IDW tool and how it works, please visit https://pro.arcgis.com/en/pro-app/latest/tool-reference/spatial-analyst/how-idw-works.htm.

Figure S1: Cumulative distribution functions for 5-year time intervals for hurricanes

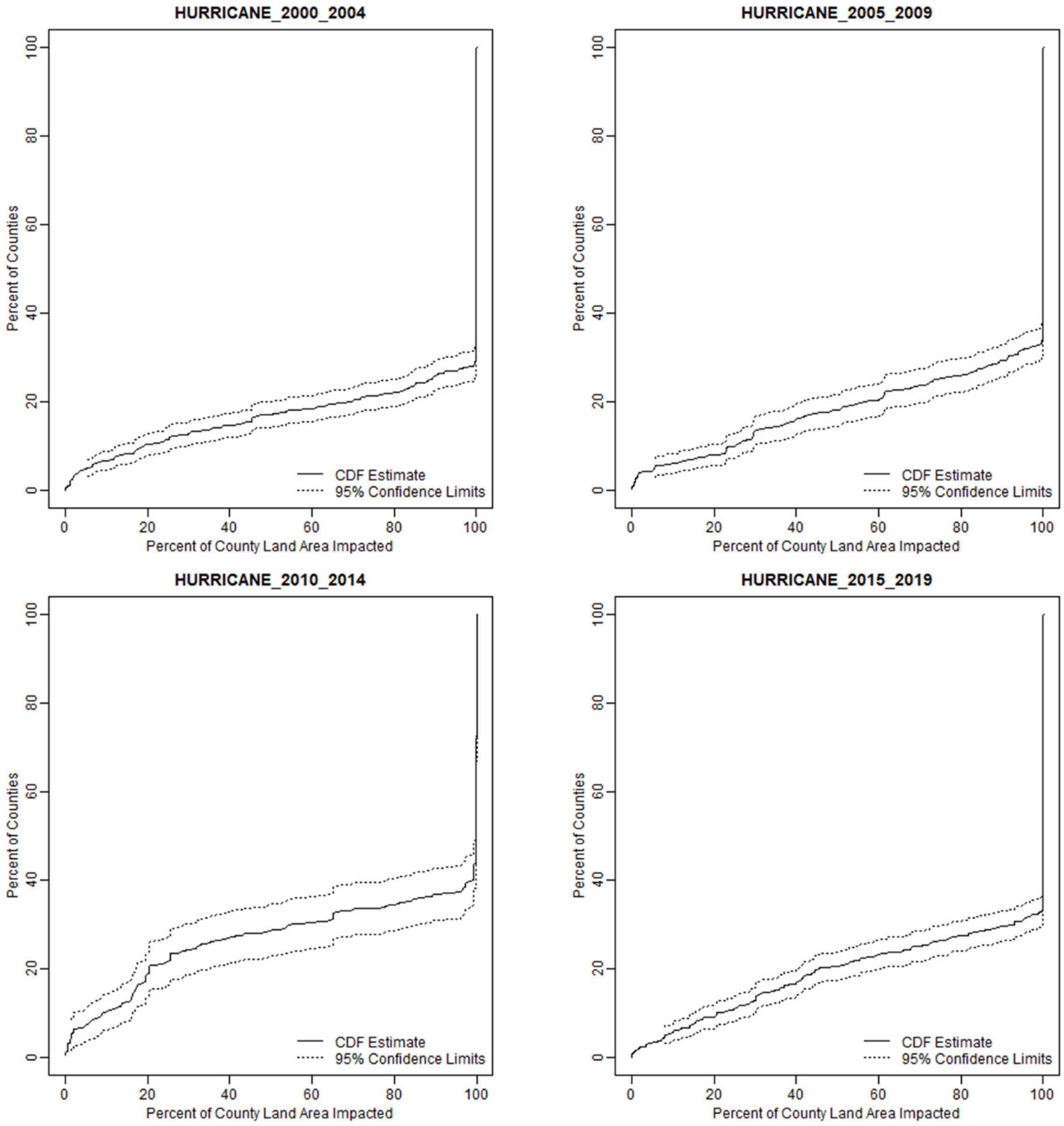

Figure S2: Cumulative distribution functions for 5-year time intervals for landslides

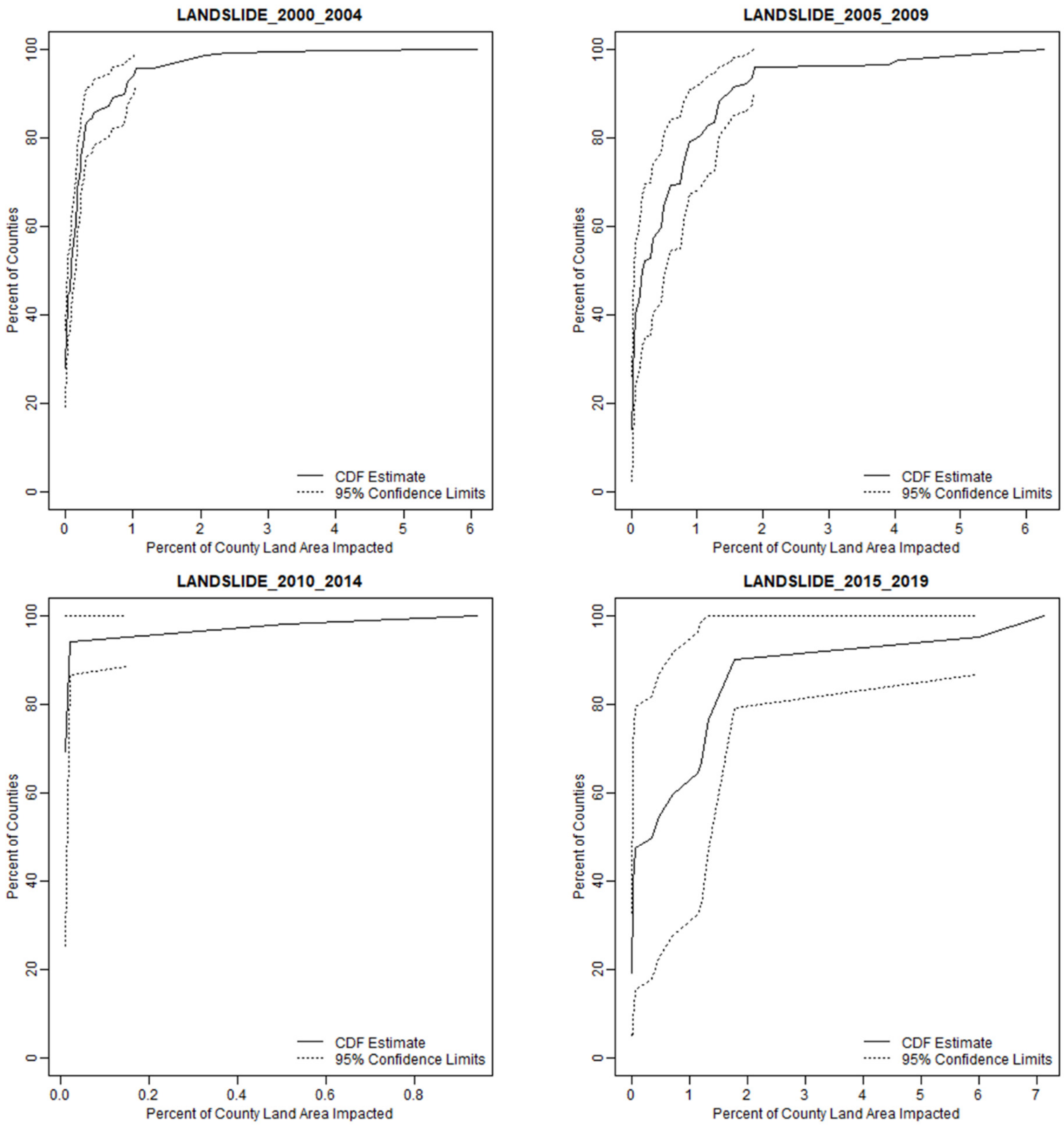

Figure S3: Cumulative distribution functions for 5-year time intervals for tornadoes

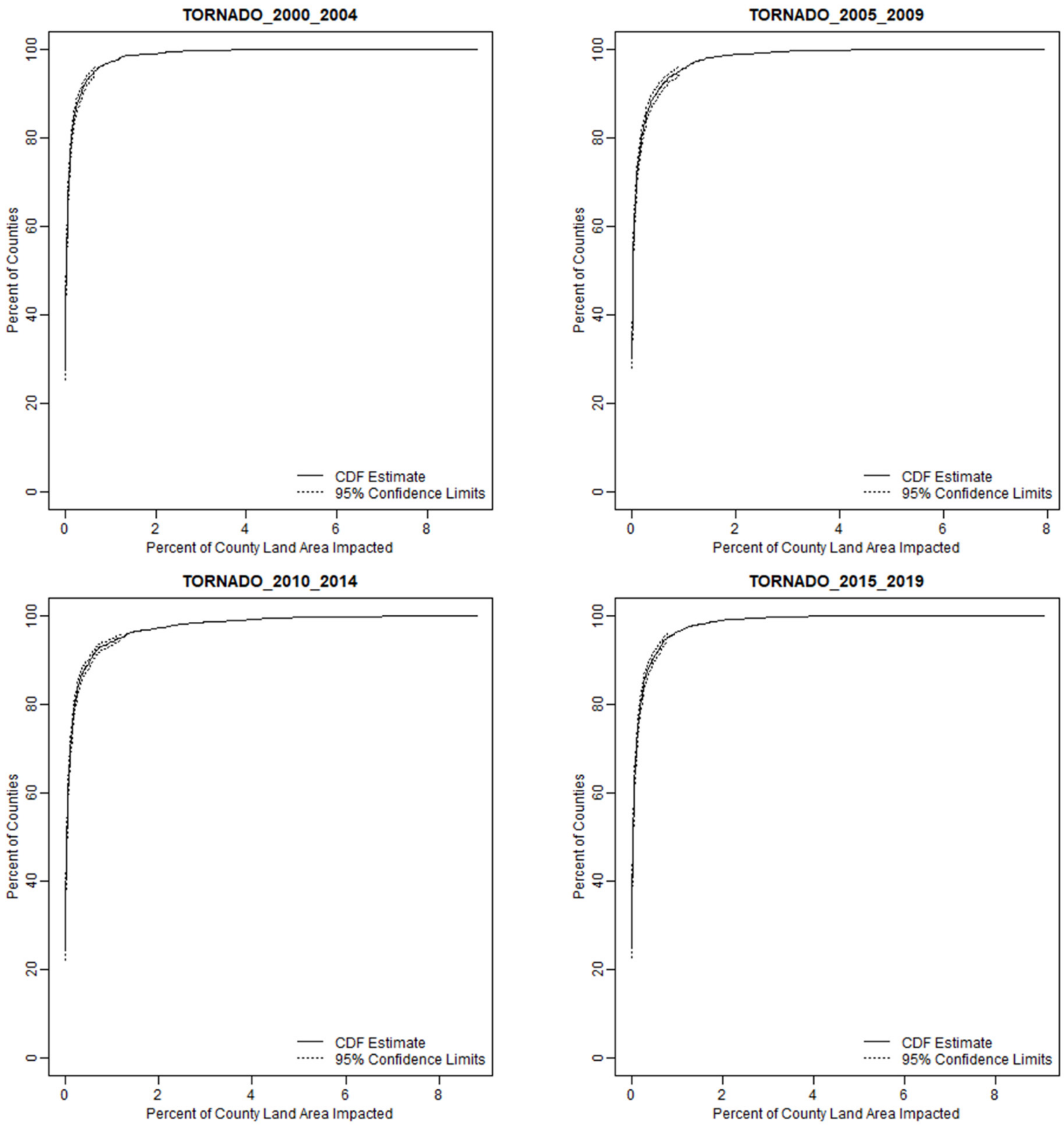

Figure S4: Cumulative distribution functions for 5-year time intervals for tropical storms

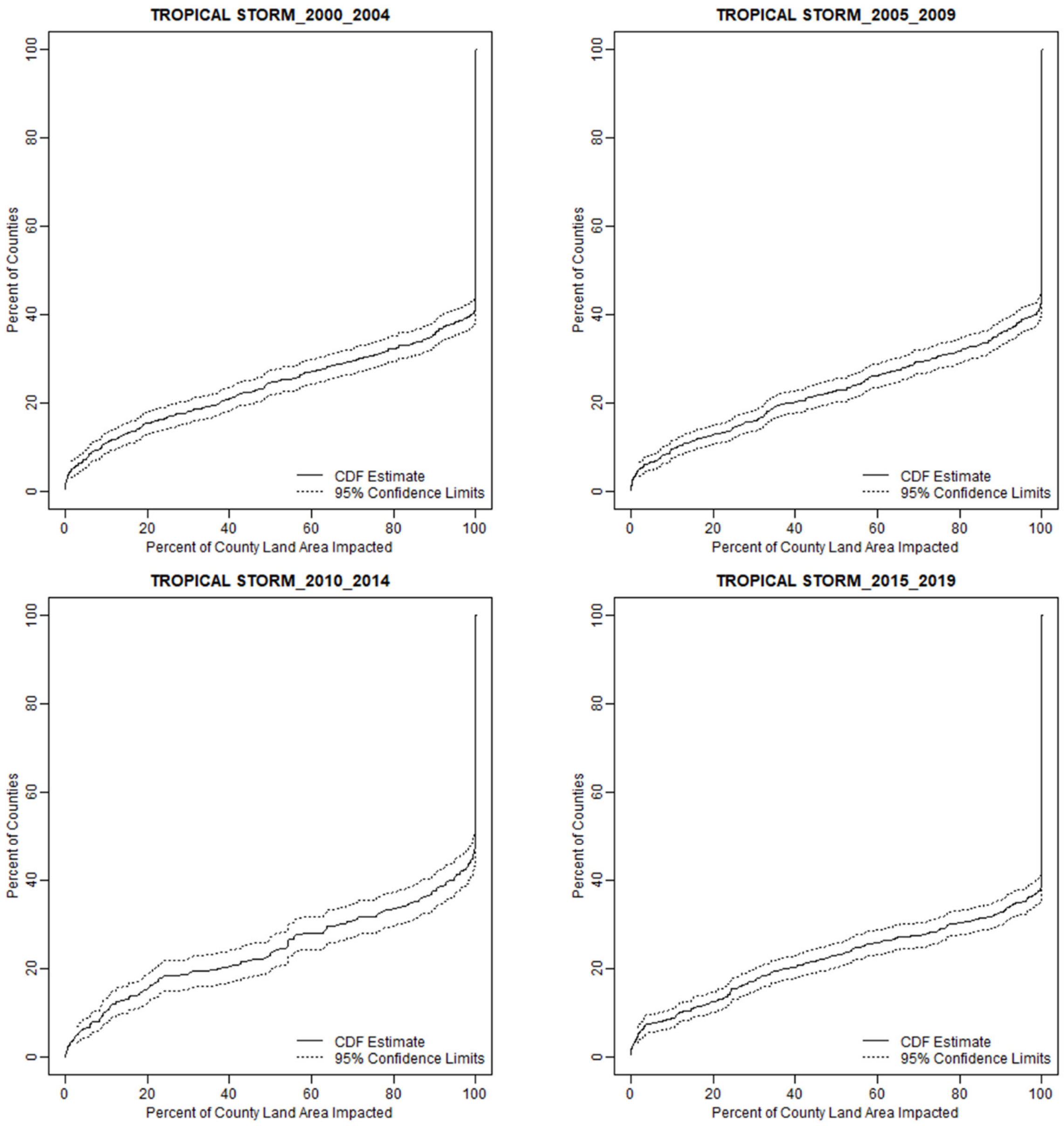

Figure S5: Cumulative distribution functions for 5-year time intervals for wildfires

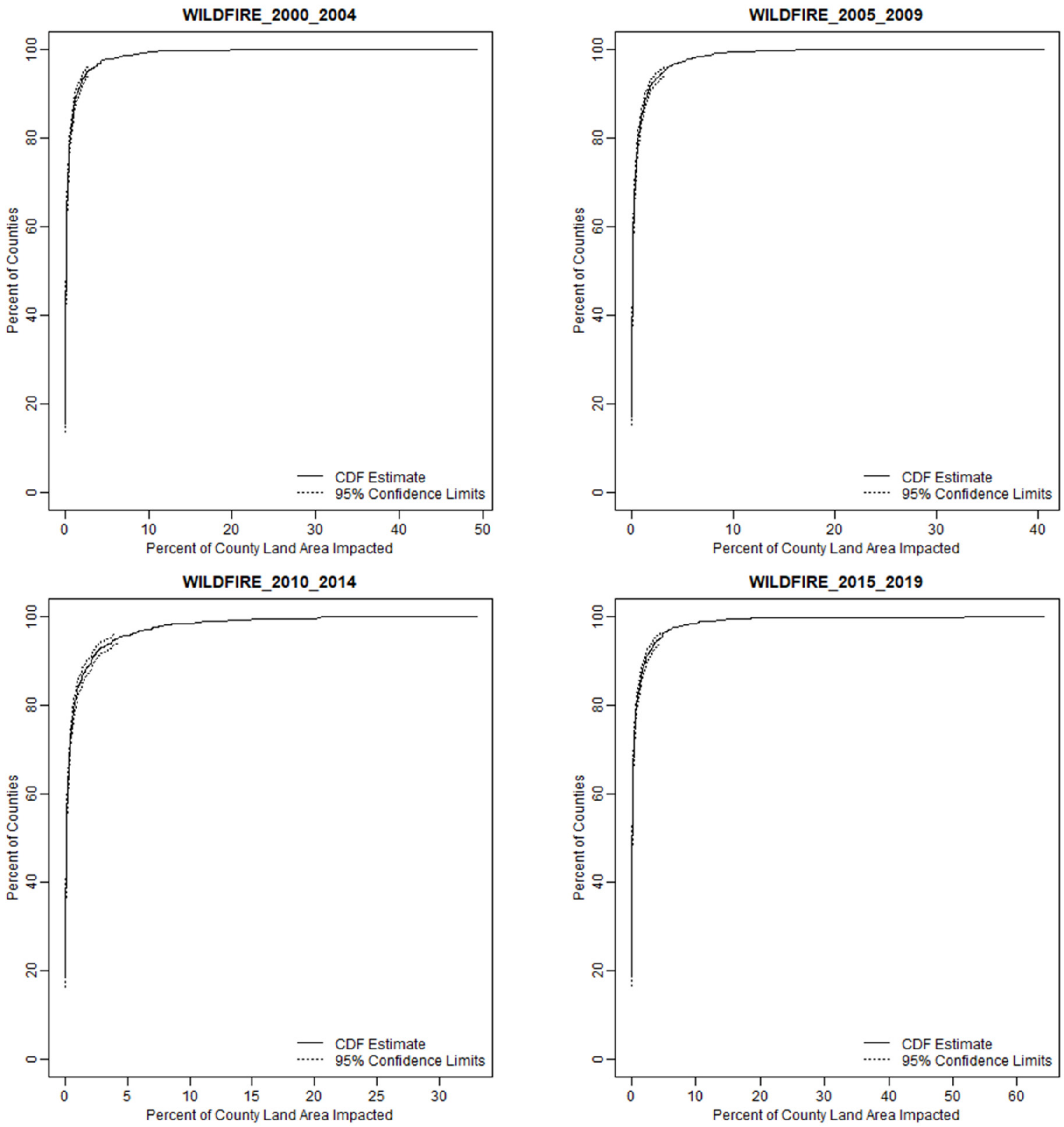

Figure S6: Cumulative distribution function for 2015-2019 for coastal flooding

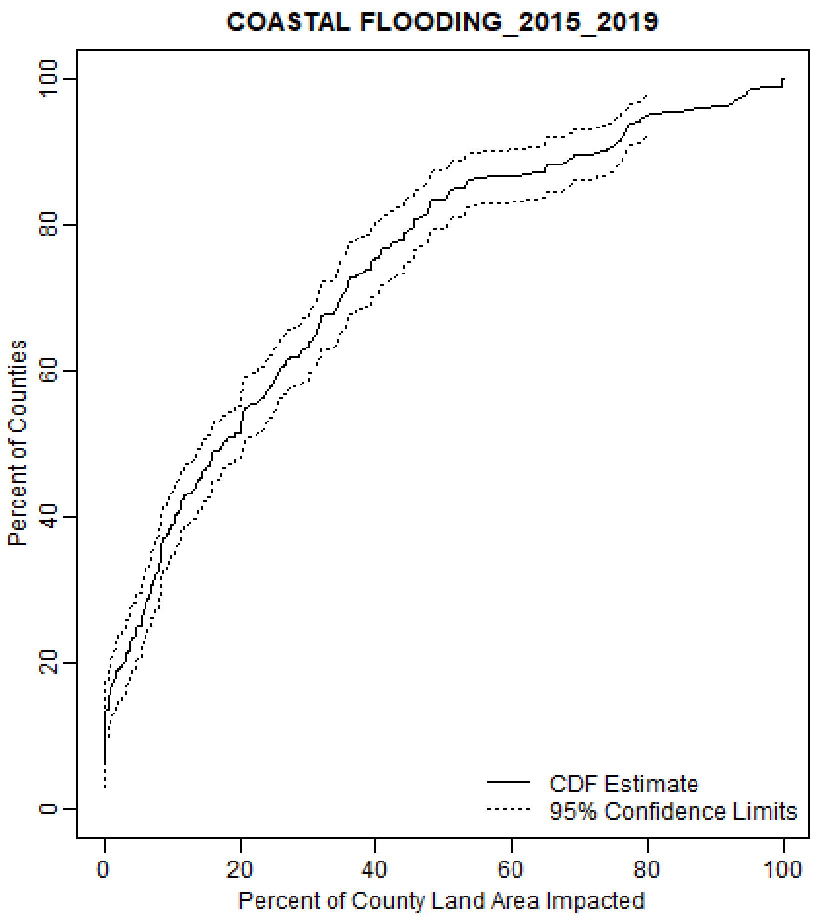

Figure S7: Cumulative distribution function for 2015-2019 for earthquakes

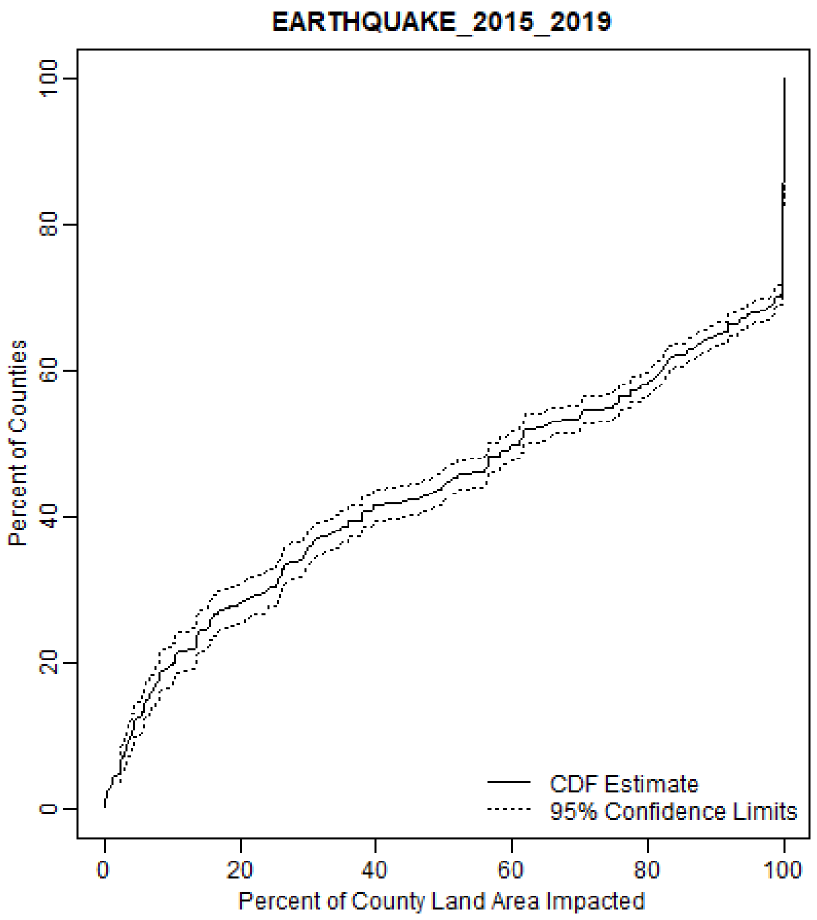

Figure S8: Cumulative distribution function for 2015-2019 for inland flooding

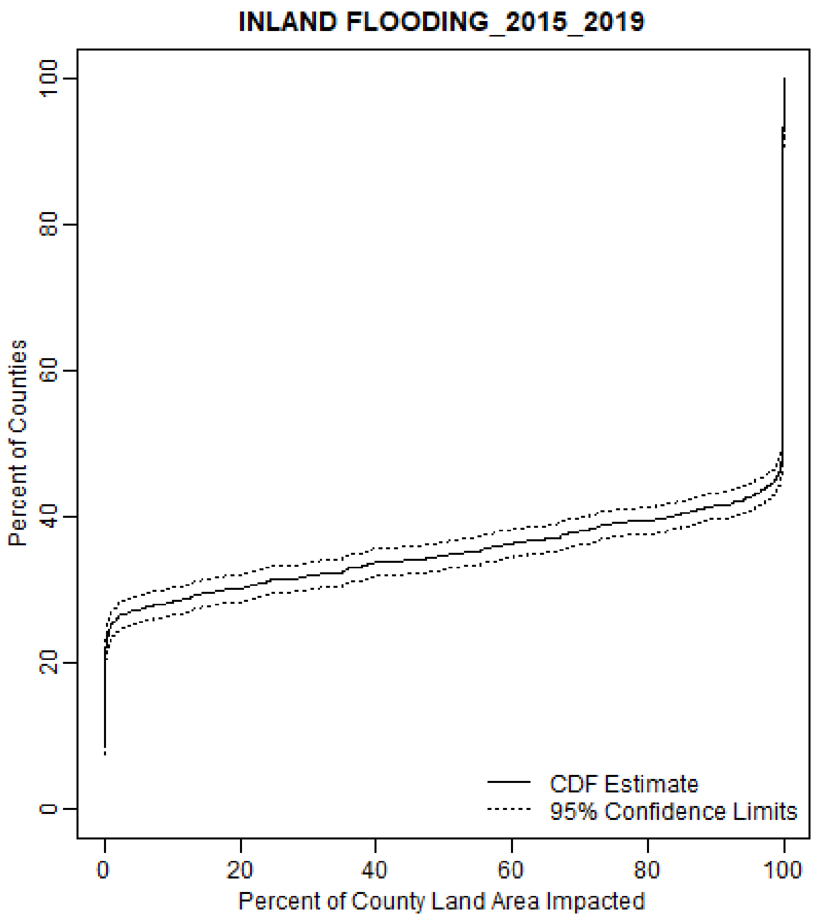

Figure S9: Spatial distributions of the incidence of hurricanes for 5-year increments: a) 2000-2004, b) 2005-2009, c) 2010-2014, d) 2015-2019

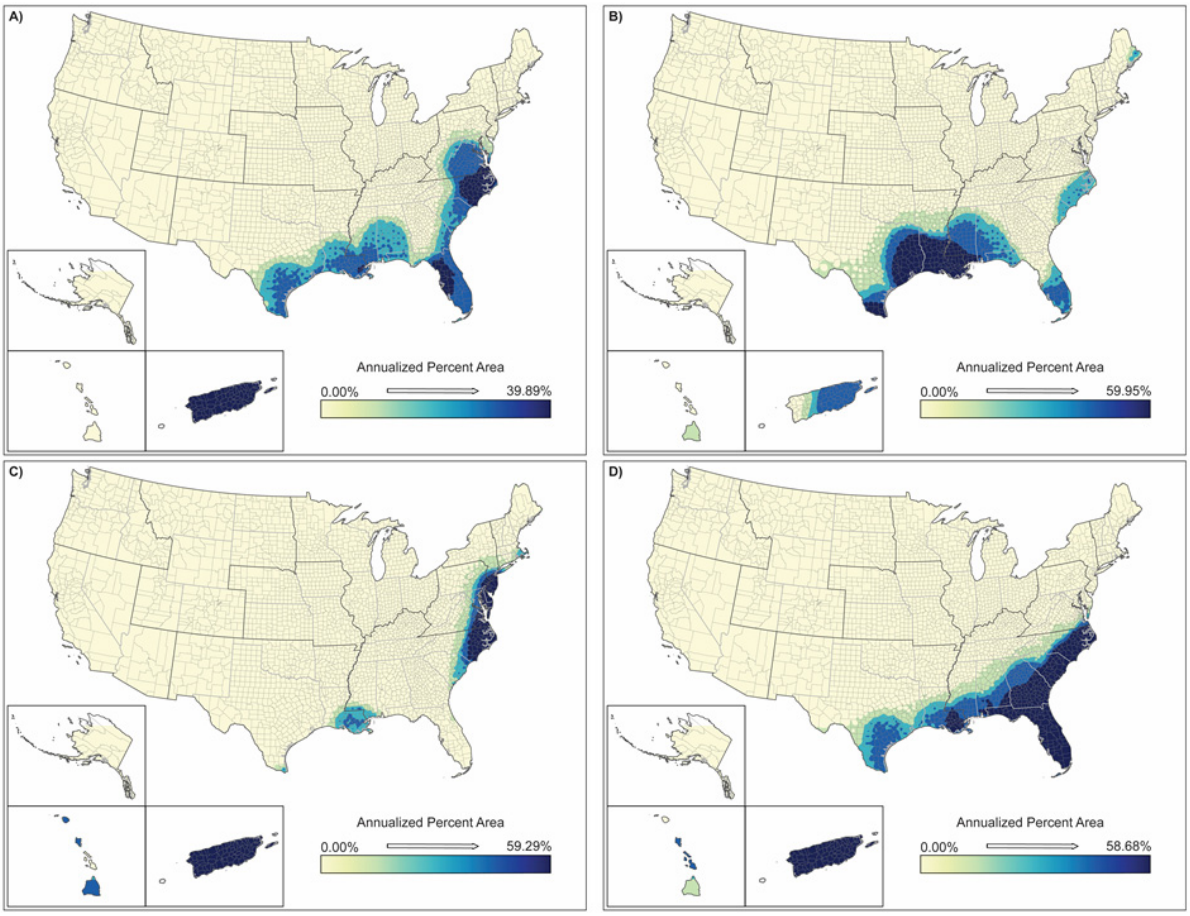

Figure S10: Trends for hurricane incidence for 2000-2019

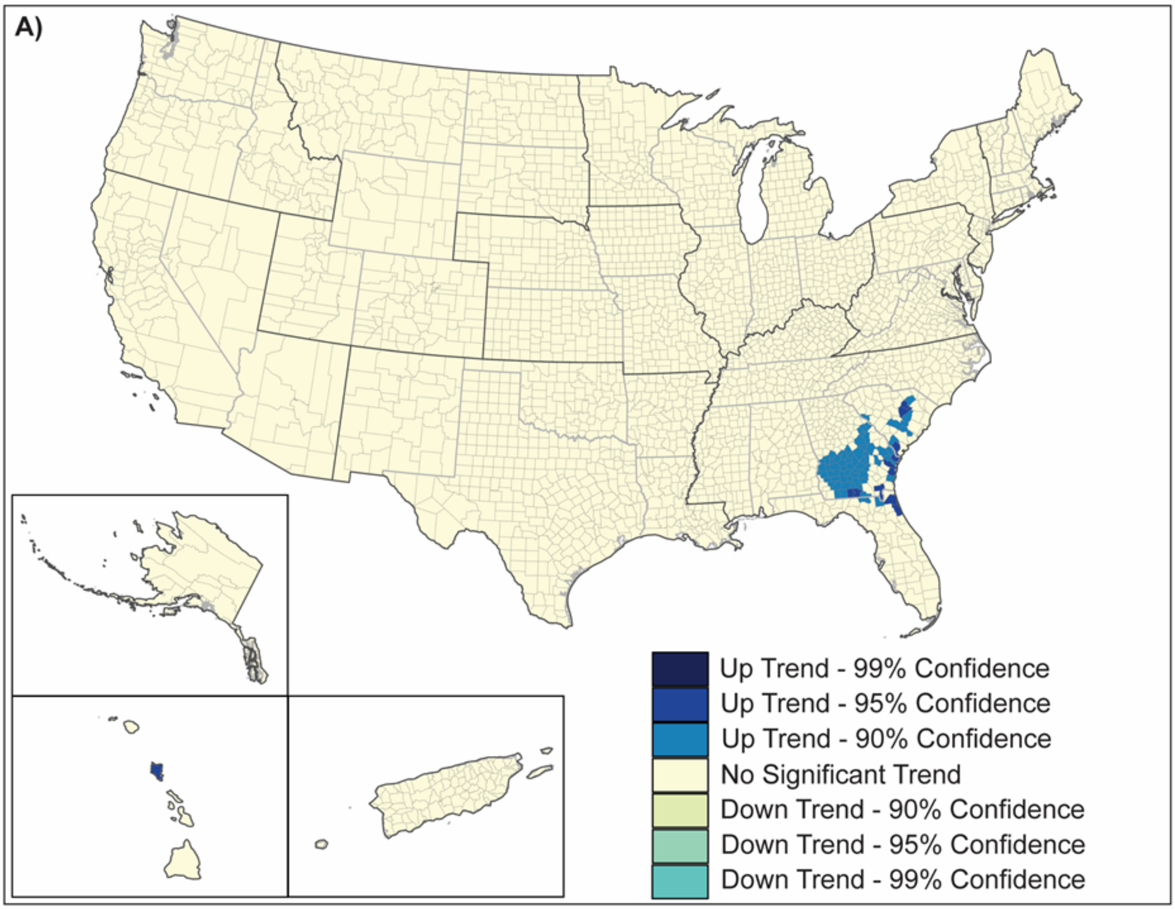

Figure S11: Spatial distributions of the incidence of tropical storms for 5-year increments: a) 2000-2004, b) 2005-2009, c) 2010-2014, d) 2015-2019

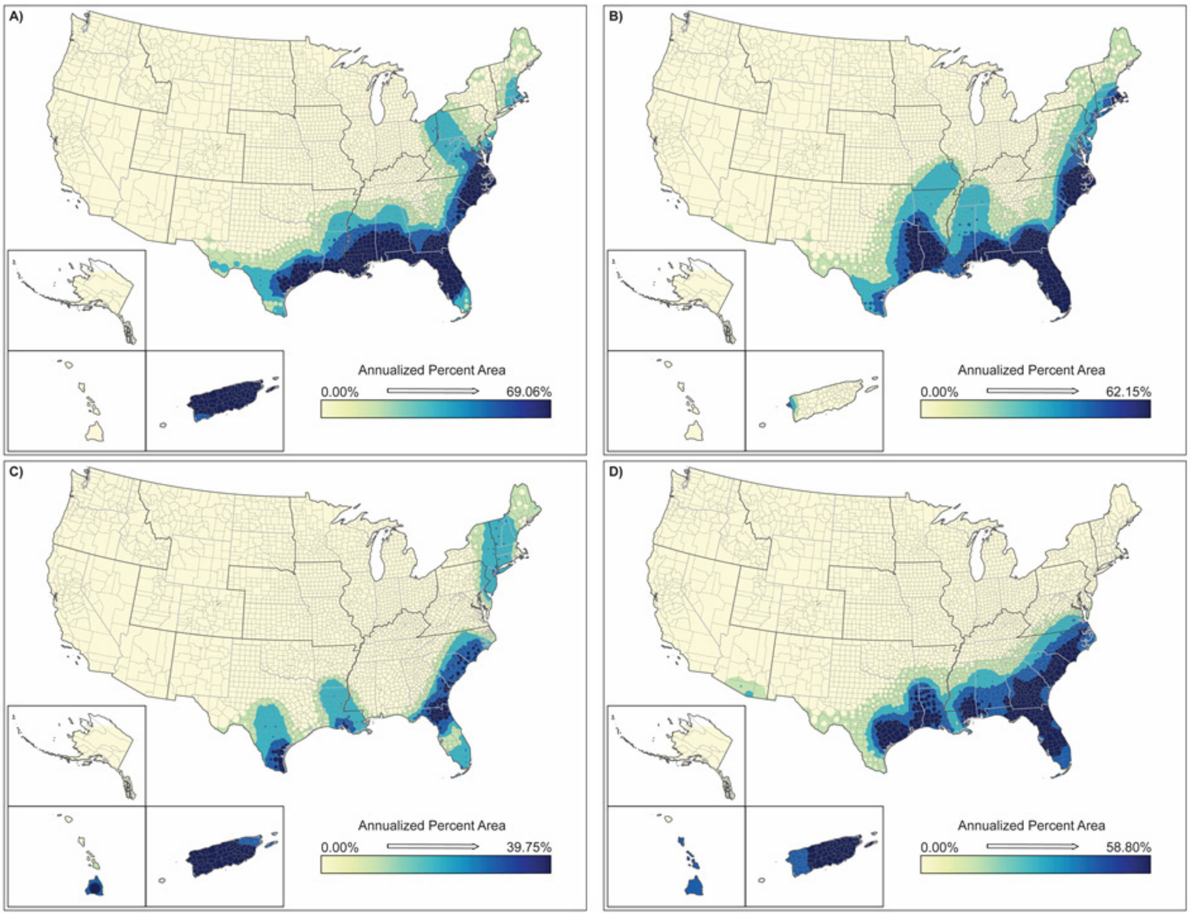

Figure S12: Trends for tropical storms incidence for 2000-2019

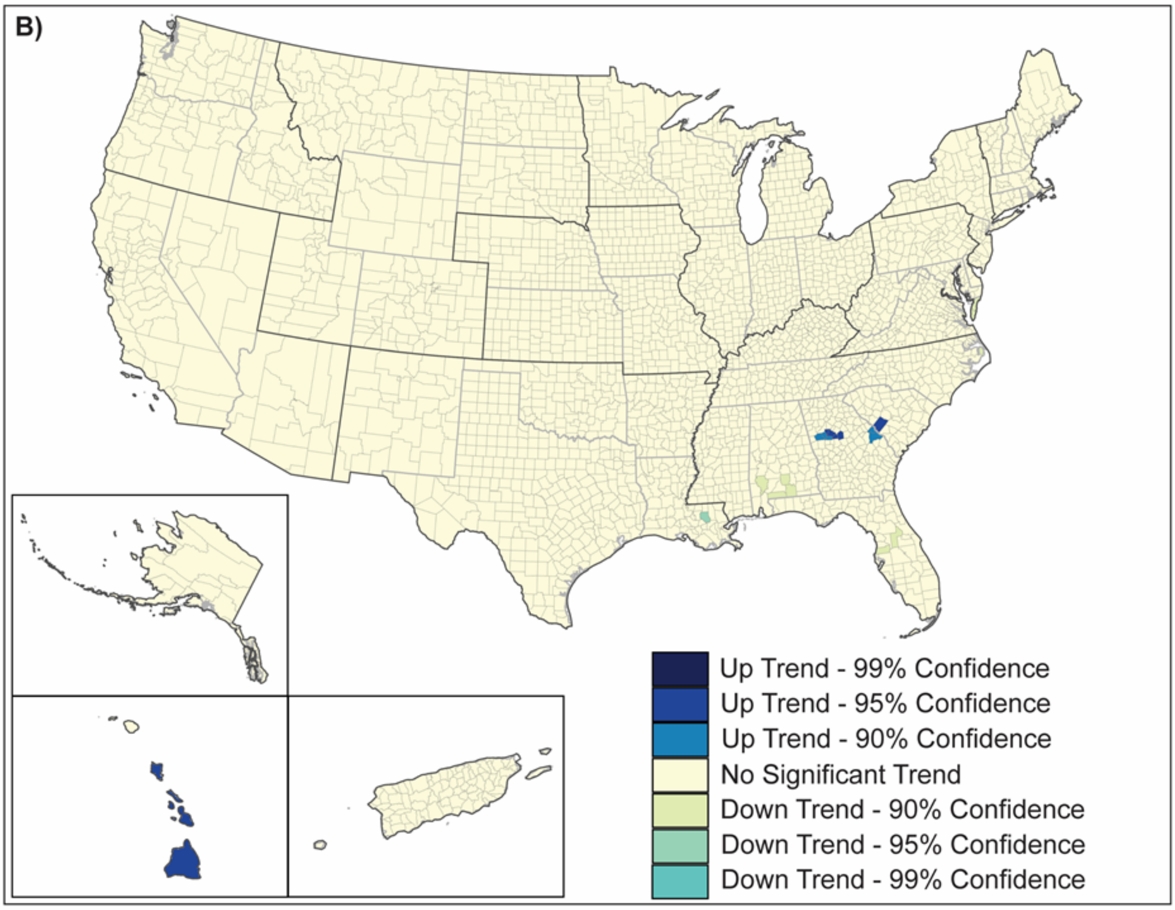

Figure S13: Spatial distributions of the incidence of wildfires for 5-year increments: a) 2000-2004, b) 2005-2009, c) 2010-2014, d) 2015-2019

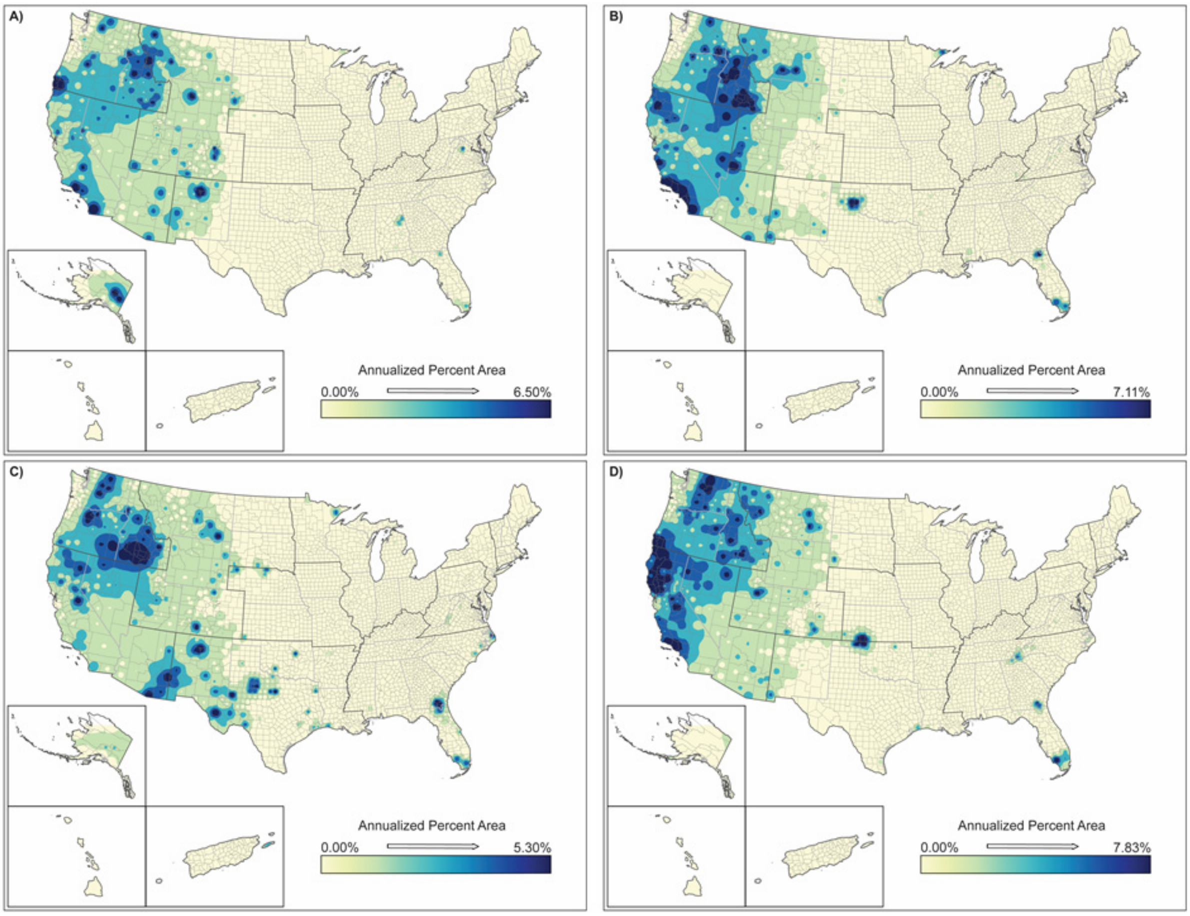

Figure S14: Trends for wildfires incidence for 2000-2019

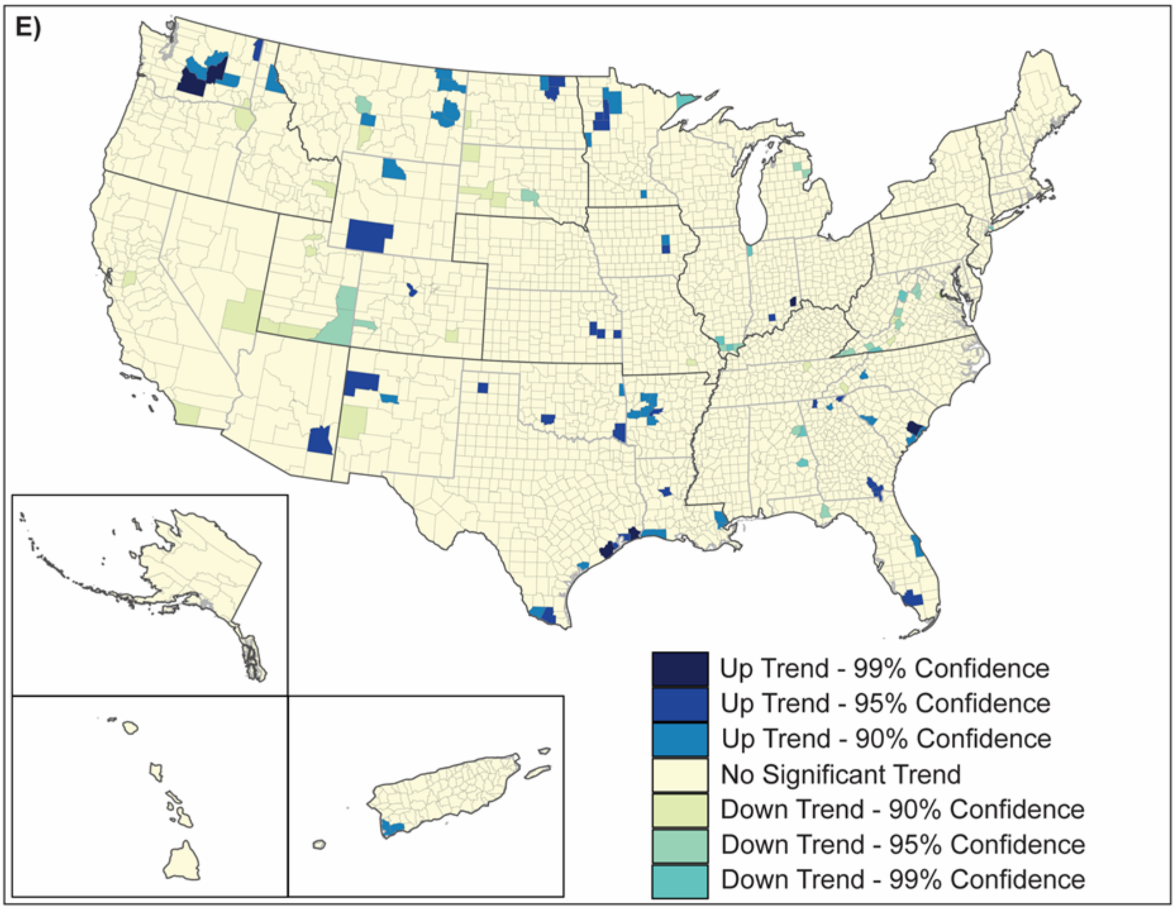

Figure S15: Spatial distributions of the incidence of drought for 5-year increments: a) 2000-2004, b) 2005-2009, c) 2010-2014, d) 2015-2019

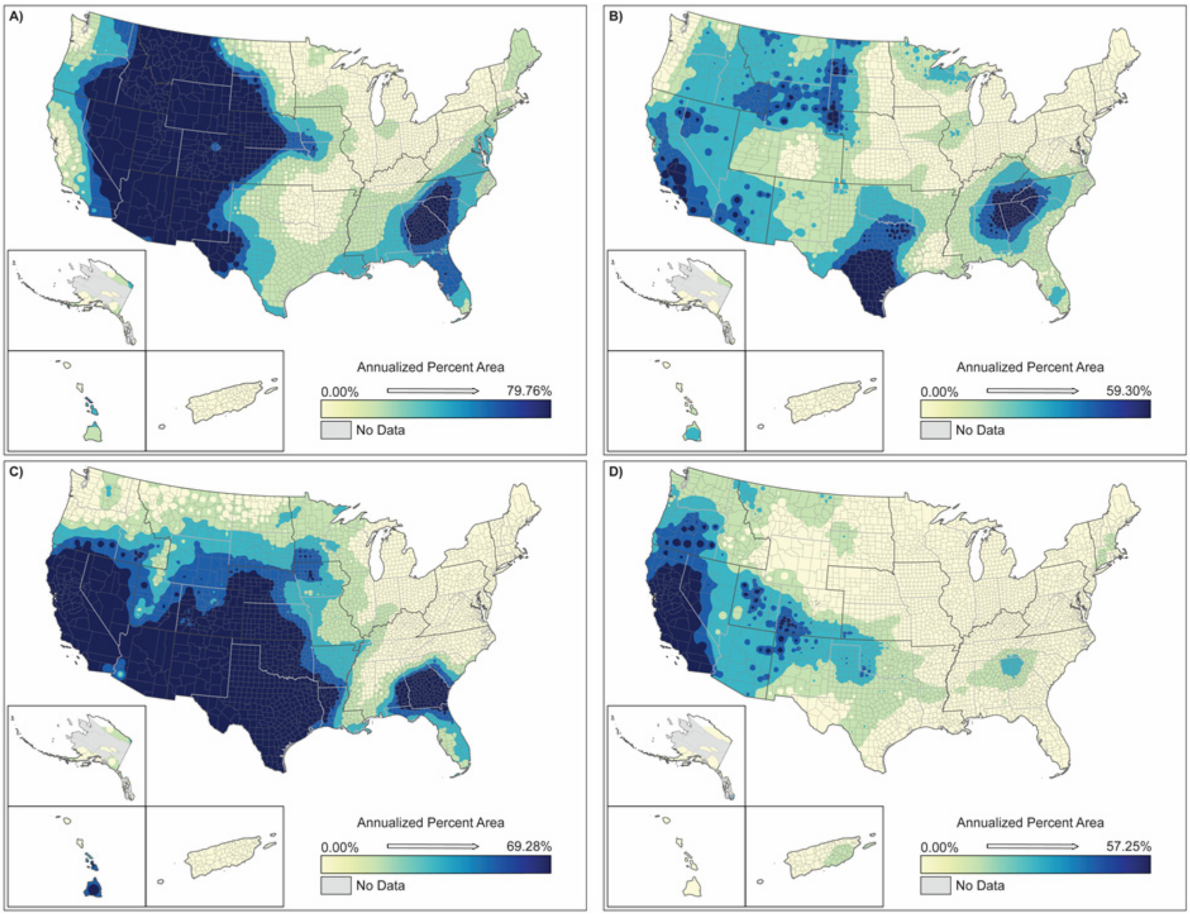

Figure S16: Trends for drought incidence for 2000-2019

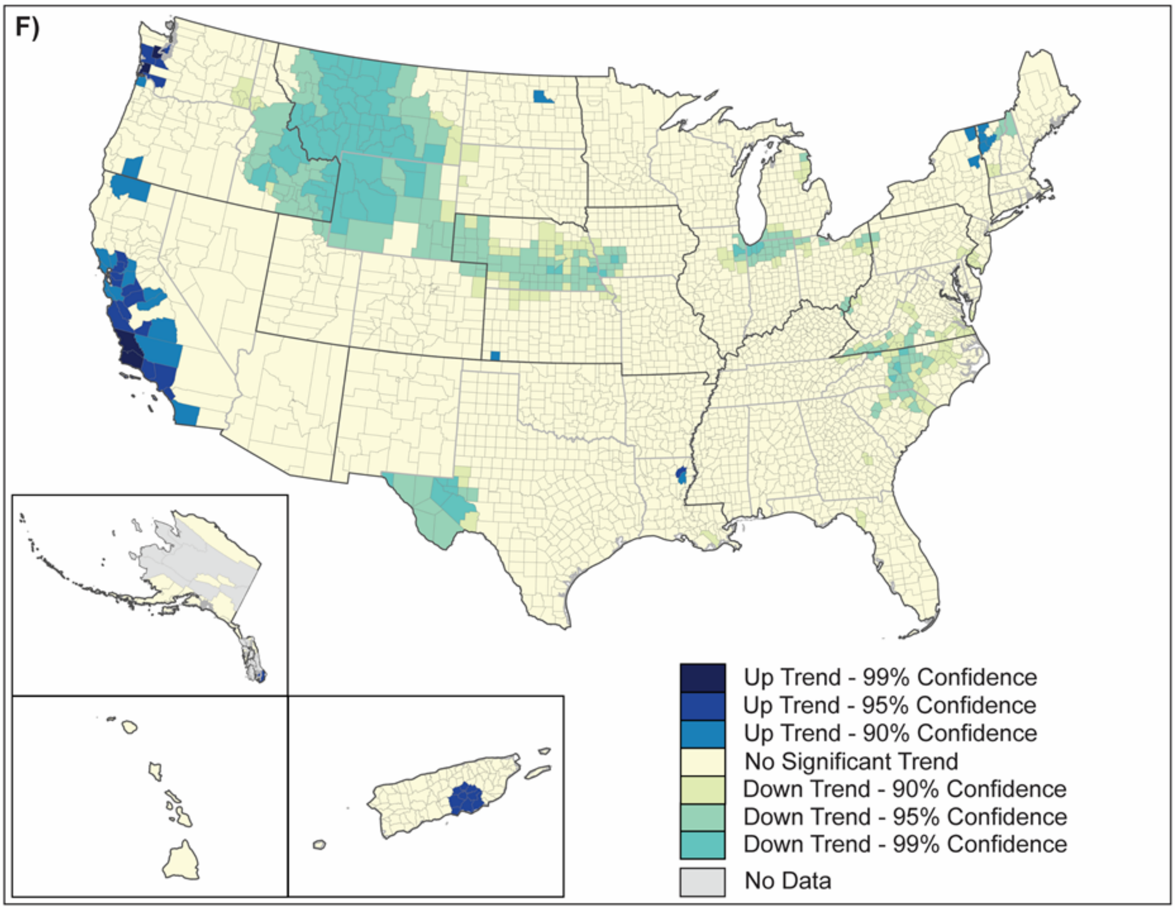

Figure S17: Spatial distributions of the incidence of landslides for 5-year increments: a) 2000-2004, b) 2005-2009, c) 2010-2014, d) 2015-2019

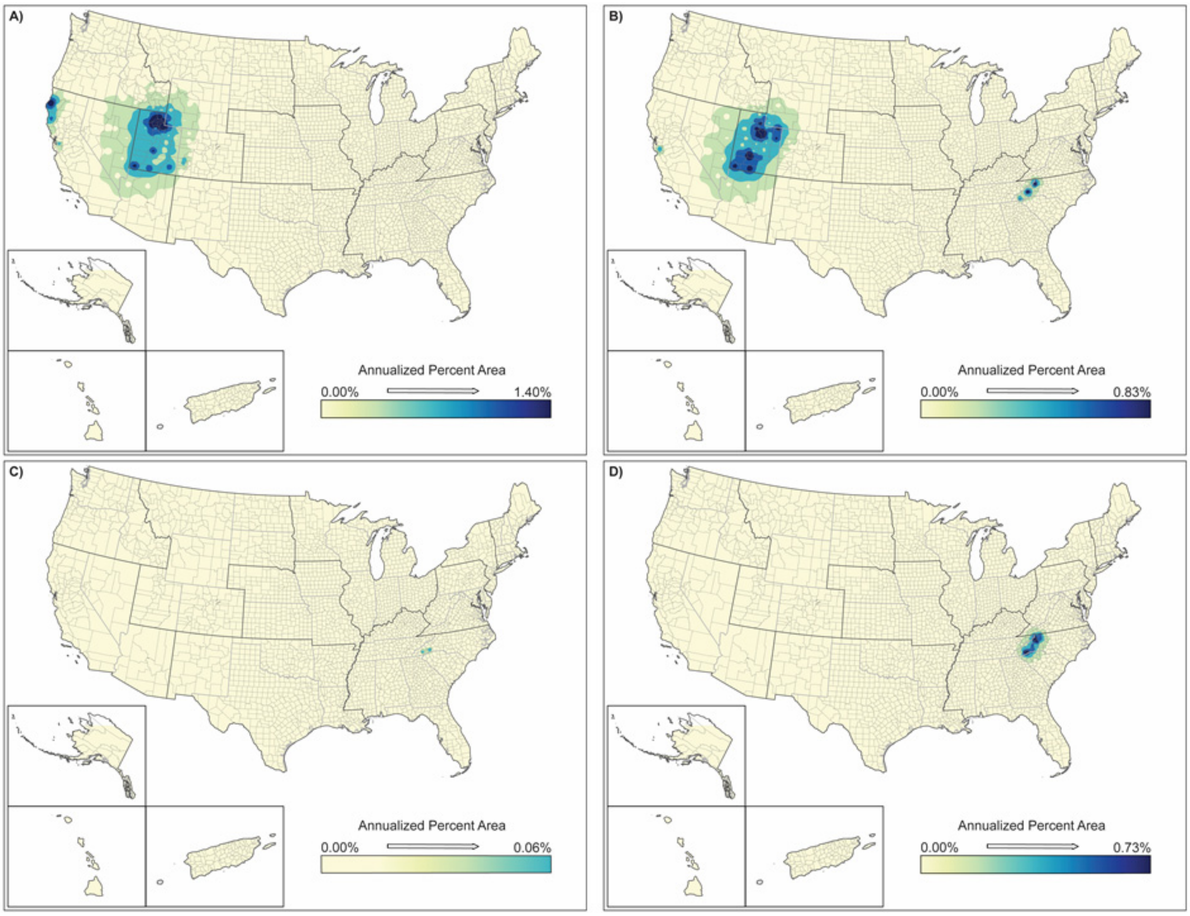

Figure S18: Trends for landslides incidence for 2000-2019

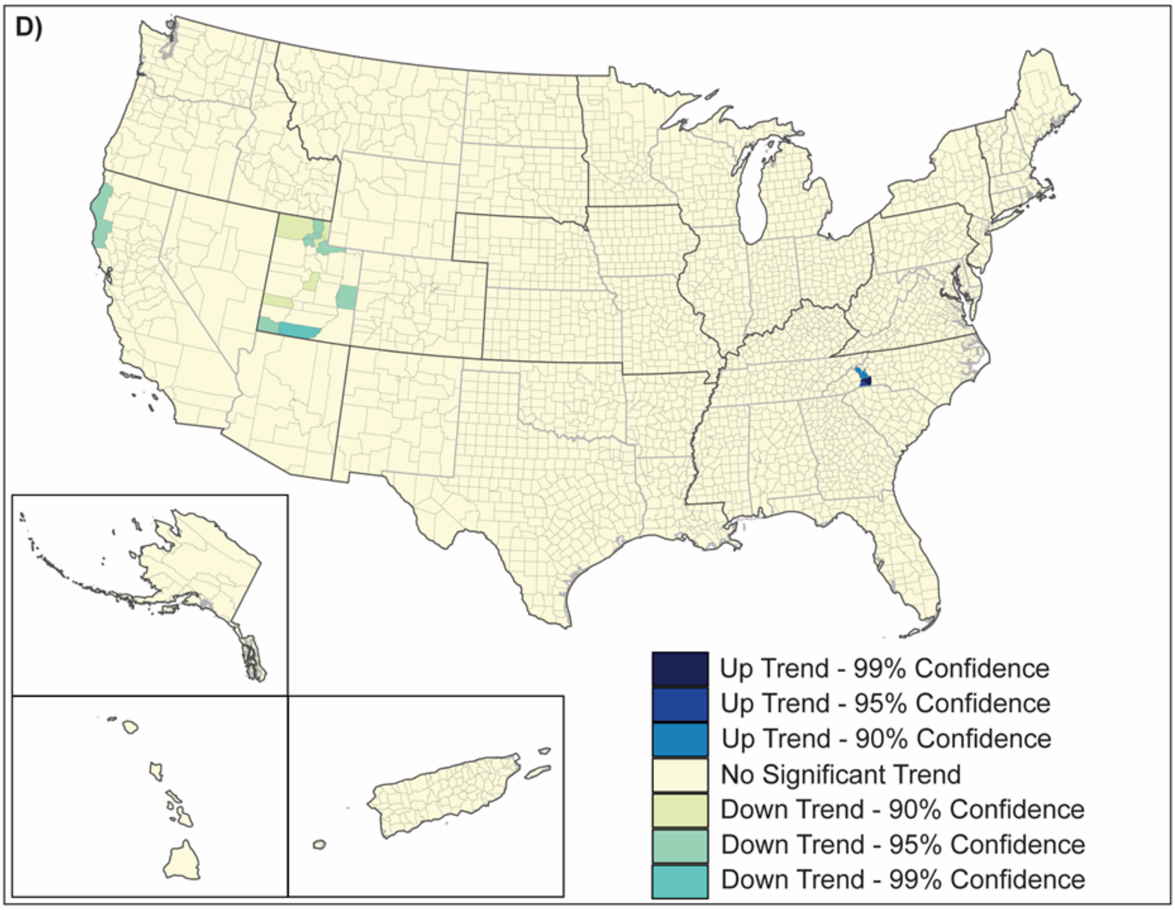

Figure S19: Spatial distributions of the incidence of tornadoes for 5-year increments: a) 2000-2004, b) 2005-2009, c) 2010-2014, d) 2015-2019

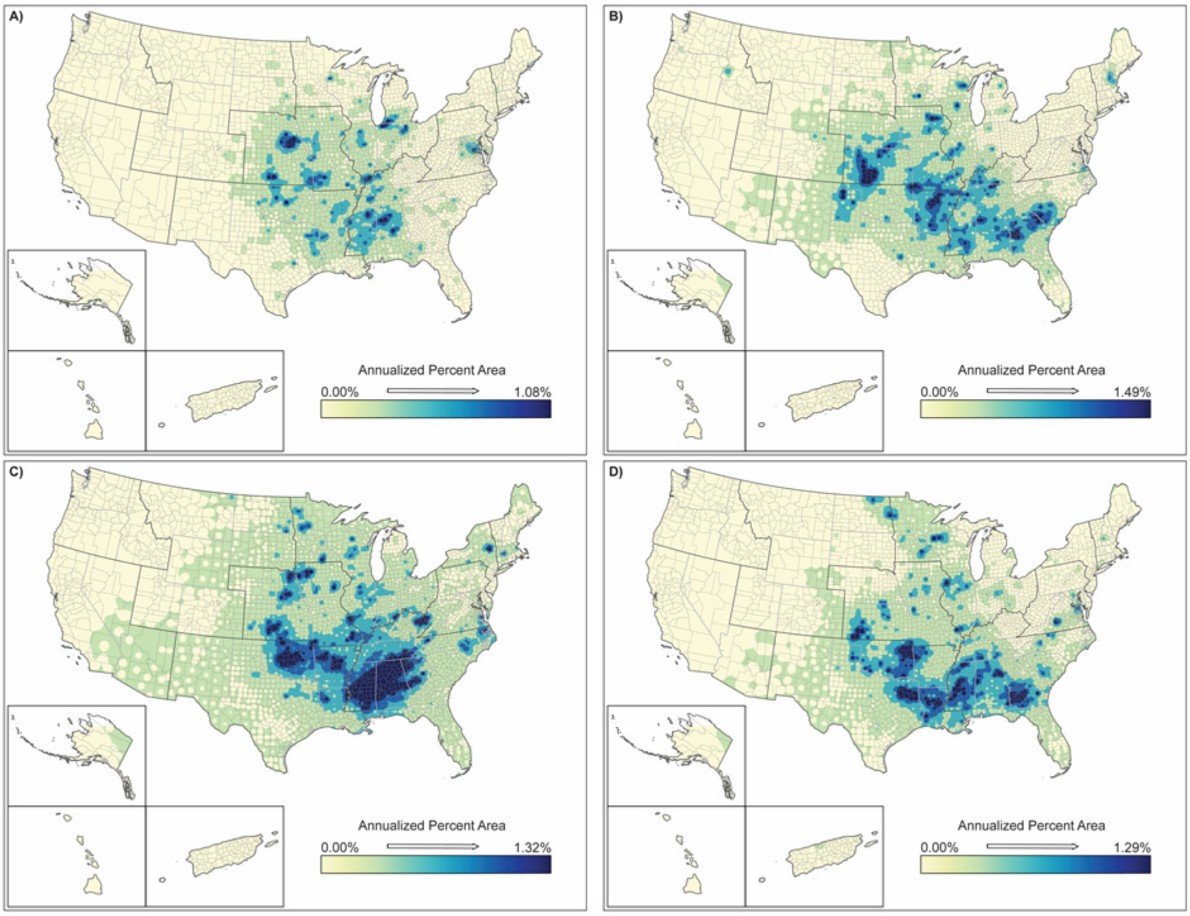

Figure S20: Trends for tornado incidence for 2000-2019

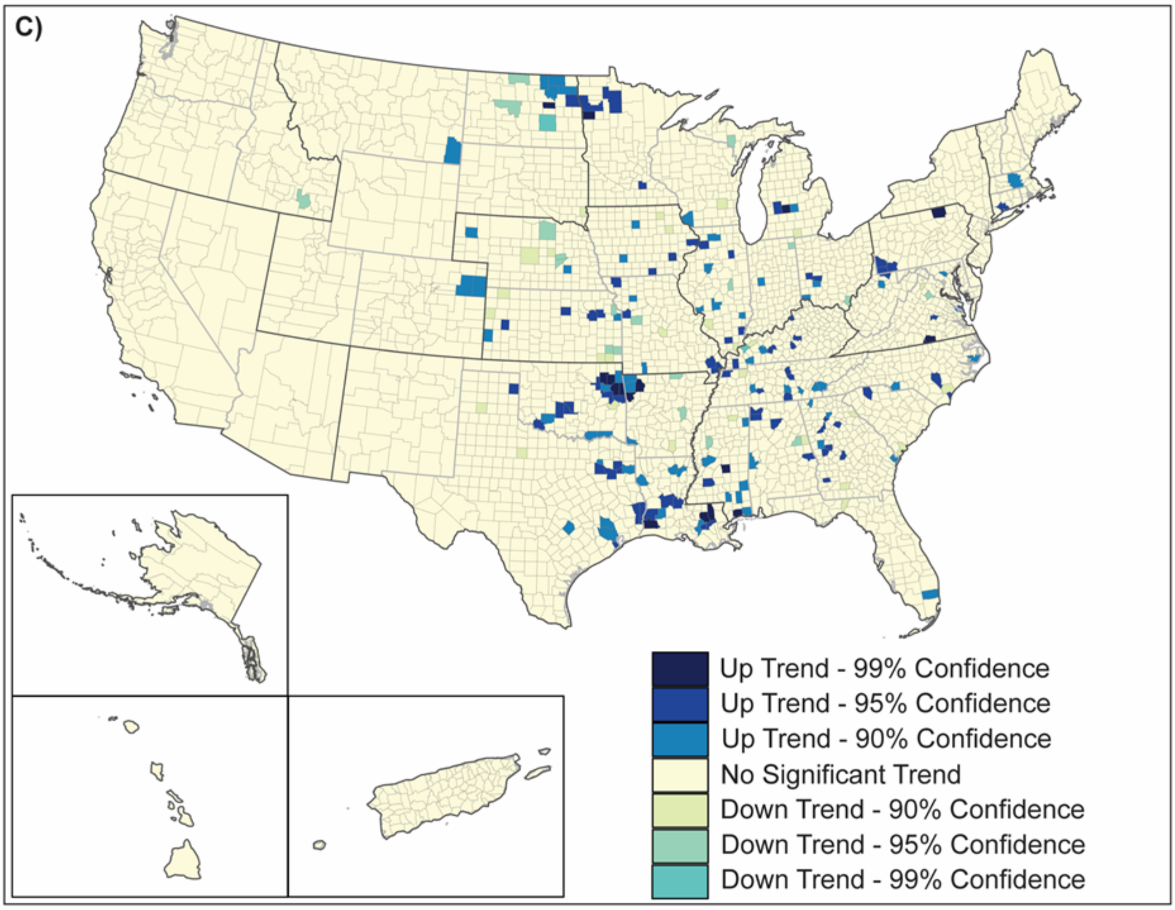

Supplement: Supplement1 [file NIHMS1831416-supplement-Supplement1.zip › sustainability-1629926-supplementary.pdf]
